# Structure–Activity Relationship Evaluation of Melatonin and Its Derivatives for Wound-Healing Applications: A Combined Network Pharmacology, Molecular Docking, and Biological Validation Approach

**DOI:** 10.3390/ijms27156699

**Published:** 2026-07-27

**Authors:** Pimolwan Siriparu, Bunleu Sungthong, Ploenthip Puthongking

**Affiliations:** 1 Graduate School, Faculty of Pharmaceutical Sciences, Khon Kaen University, Khon Kaen 40002, Thailand; pimolwan.s@kkumail.com; 2 Integrative Pharmaceuticals and Innovation of Pharmaceutical Technology Research Unit, Faculty of Pharmacy, Mahasarakham University, Maha Sarakham 44150, Thailand; bunleu.s@msu.ac.th; 3 Division of Pharmaceutical Chemistry, Faculty of Pharmaceutical Sciences, Khon Kaen University, Khon Kaen 40002, Thailand

**Keywords:** *N*-amide derivative, hemostasis, inflammation, proliferation, remodeling, HIF-1, NHDF, wound healing

## Abstract

Non-healing wounds remain a clinical challenge due to their complex pathophysiology and limited therapeutic options. These conditions are driven by complex molecular mechanisms, including inflammation, cell proliferation, and tissue remodeling. Melatonin (MLT) and its *N1*- and *N2*-substituted derivatives are known to exhibit potent antioxidant and anti-inflammatory properties; however, their specific therapeutic mechanisms in wound healing remain largely unexplored. Therefore, this study aimed to investigate the potential wound-healing properties of MLT and its six derivatives using an integrated computational and in vitro validation approach. Potential targets of MLT and its derivatives were screened using SwissTargetPrediction (version 2023 release) and SuperPred (version 3.0), yielding 491 candidate targets. These targets were cross-referenced with the GeneCards (version 5.24.0) database to map their involvement across the four phases of wound healing: hemostasis, inflammation, proliferation, and remodeling. Network interaction models were constructed using Cytoscape (version 3.10.3) and GeneMANIA (version 3.6.0), and pathway enrichment was analyzed using the ShinyGO (version 0.85.1) platform. Enrichment analysis prioritized HIF-1-related signaling as a candidate regulatory axis associated with the predicted targets of melatonin derivatives across the inflammatory, proliferative, and remodeling phases of wound healing. In vitro validation using normal human dermal fibroblasts (NHDFs) demonstrated that all compounds, at non-toxic concentrations, significantly enhanced cell viability, as measured by the MTT assay. Furthermore, wound scratch assays revealed that the *N2*-bromobenzoyl-substituted derivative (EBMLT) accelerated cell migration, achieving complete wound gap closure within 24 h and outperforming the parent compound. Molecular docking simulations using AutoDock 4.2 predicted favorable binding interactions of the derivatives toward key wound healing-related targets (NF-κB, EGFR, VEGFR-1, MMP-1, and MMP-13). Aromatic-substituted derivatives (BMLT, BBMLT, and EBMLT) exhibited more favorable predicted binding interactions than the parent compound across all targets, whereas the aliphatic-substituted derivative (SMLT) showed weaker predicted interactions, particularly with VEGFR-1. These findings suggest that *N1*- and *N2*-aromatic substitutions are associated with more favorable binding interactions. Notably, the *N2*-bromobenzoyl derivative (EBMLT) exhibited the most potent wound-closure activity, highlighting it as a candidate compound for wound-healing applications.

## 1. Introduction

Melatonin (*N*-acetyl-5-methoxytryptamine: MLT) is an endogenous compound with antioxidant and anti-inflammatory properties that has attracted interest for wound-healing applications. MLT has been reported to modulate the production of pro-inflammatory cytokines [1], thereby promoting the resolution of the inflammatory phase and facilitating wound healing [2,3]. Successful wound healing requires progression from the inflammatory phase to cell proliferation and tissue remodeling in order to restore normal skin structure [4,5,6]. However, this cascade is frequently delayed in patients with diabetes mellitus, as chronic wounds remain stalled in a prolonged inflammatory state and fail to progress through the physiological healing timeline [7,8].

Numerous studies have reported that MLT affects multiple processes involved in wound healing through its diverse biological activities. For instance, its antioxidant and anti-inflammatory properties have demonstrated potential for the treatment of damaged gingival tissue associated with chronic inflammation by reducing MMP-1 expression and elevating tissue inhibitor of metallopeptidase-1 (TIMP-1) level, resulting in increased collagen deposition [9]. Furthermore, topical administration of an MLT-loaded hydrogel in a full-thickness rat wound model resulted in faster wound closure than untreated controls. This therapeutic efficacy was closely linked to enhanced angiogenesis and vascular reactivity, accelerated granulation, re-epithelialization, and collagen deposition, suggesting that MLT may promote the proliferative phase of wound healing [7].

Intriguingly, structural modification of MLT seems to enhance its biological activities, particularly through substitution at its *N1* or *N2* positions [10,11,12,13,14,15,16]. These modifications mimic key pharmacophoric features of indomethacin, a well-known nonsteroidal anti-inflammatory drug (NSAID) that acts as a potent cyclooxygenase (COX) inhibitor [17], which possesses a core structure with an indole ring coupled with an aromatic group and 4-chlorobenzoyl moiety at *N1*-position. Our previous studies demonstrated that MLT derivatives with aromatic or aliphatic substituents at the *N1*-position exhibited greater antioxidant and anti-inflammatory activities than the parent compound [10,11,12,13,14,15,16]. These elevated properties suggest that such derivatives hold great promise for wound healing. Therefore, the current study utilized a network pharmacology approach to identify key molecular targets, biological functions, and biochemical pathways, with a specific focus on the HIF-1 signaling cascade and associated inflammatory and remodeling nodes governing the wound-healing effects of MLT and its derivatives. Subsequently, molecular docking simulations were performed to investigate the structure–activity relationships (SAR) of the compounds with selected wound-healing targets, followed by in vitro cell proliferation and wound scratch assays using normal human dermal fibroblasts (NHDFs) to validate our computational predictions.

## 2. Results and Discussion

### 2.1. Potential Target Genes of MLT and Its Derivatives

**Figure 1:**
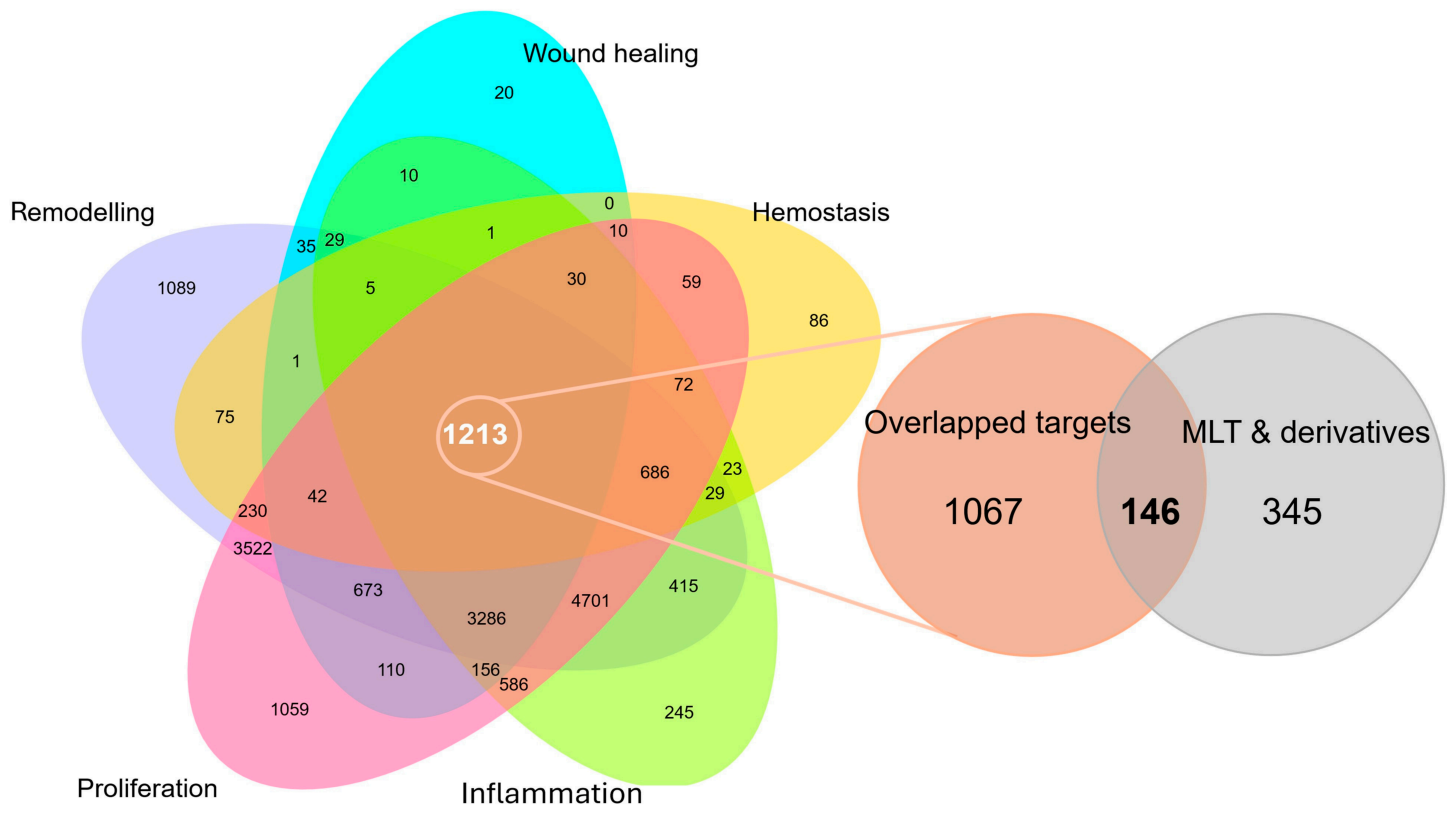
The 1213 overlapped protein on wound healing-related target from GeneCard and the 146 probable targets of MLT and derivatives on wound healing.

Target proteins associated with wound healing processes were retrieved from the GeneCards database using the keywords: wound healing, hemostasis, inflammation, proliferation, and remodeling. The Venn diagram identified 1213 overlapping target proteins (Figure 1). Target prediction of MLT and its derivatives identified 184 proteins using SuperPred and 491 proteins using SwissTargetPrediction based on SMILES structures. Comparison of the predicted targets with wound healing-related proteins identified 146 overlapping target proteins (Figure 1).

### 2.2. Protein–Protein Interaction of MLT and Derivatives on Wound Healing

**Figure 2:**
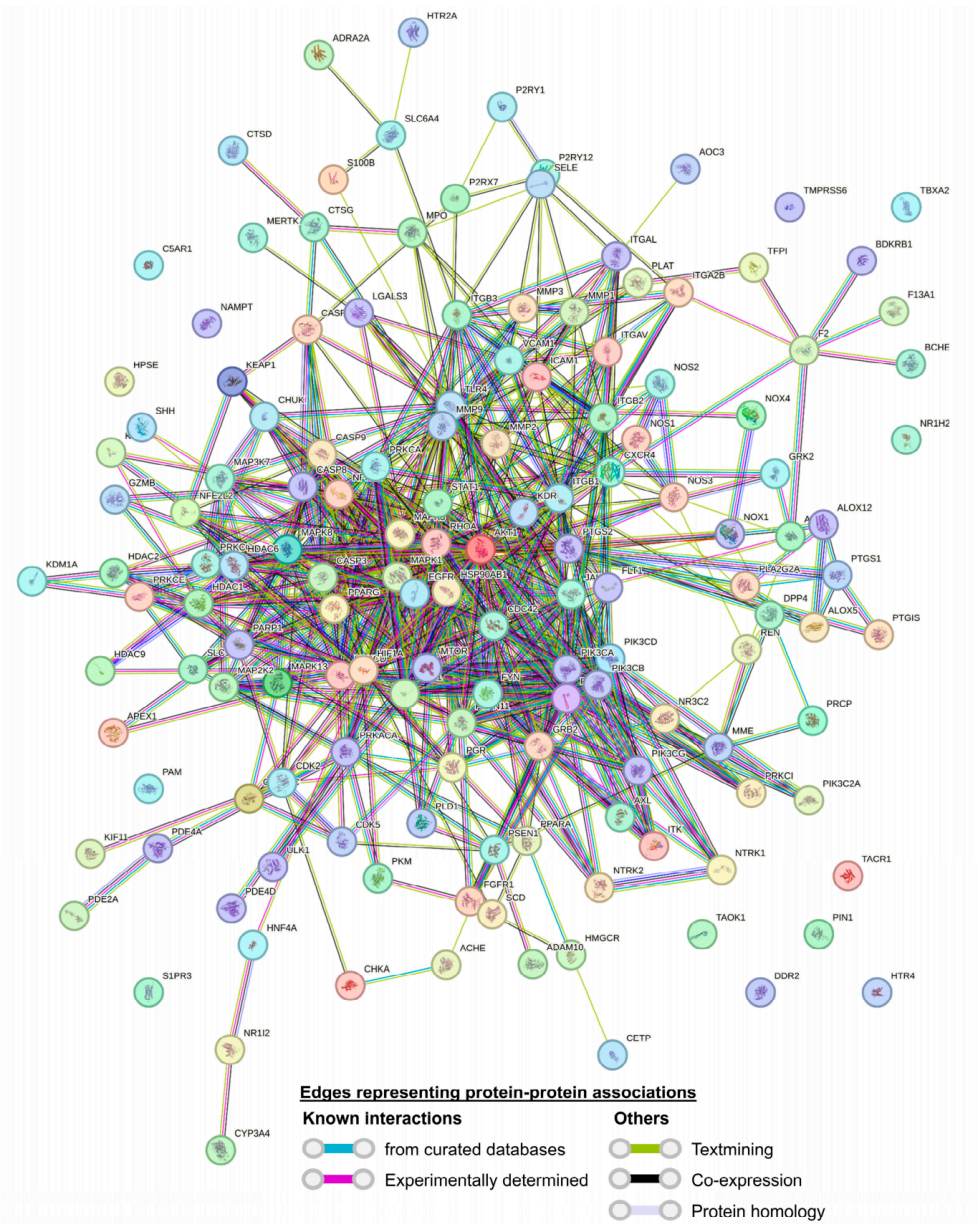
The PPI of 146 probable targets from MLT and derivatives on wound healing.

Protein–protein interaction (PPI) analysis showed that the 146 overlapping target proteins were closely interconnected. The PPI network consisted of 145 nodes and 672 edges, with an average node degree of 9.27, an average local clustering coefficient of 0.464, an expected number of edges of 209, and a PPI enrichment *p*-value less than 1 × 10^−16^ (Figure 2). AKT (RAC-alpha serine/threonine-protein kinase) exhibited the highest degree (48 edges), followed by EGFR (epidermal growth factor receptor; 45 edges), MAPK3 (mitogen-activated protein kinase 3; ERK1; 32 edges), HIF1A (hypoxia-inducible factor 1-alpha; 30 edges), and NFKB1 (Nuclear factor NF-kappa-B p105 subunit; 30 edges). These proteins were considered key target proteins.

**Figure 3:**
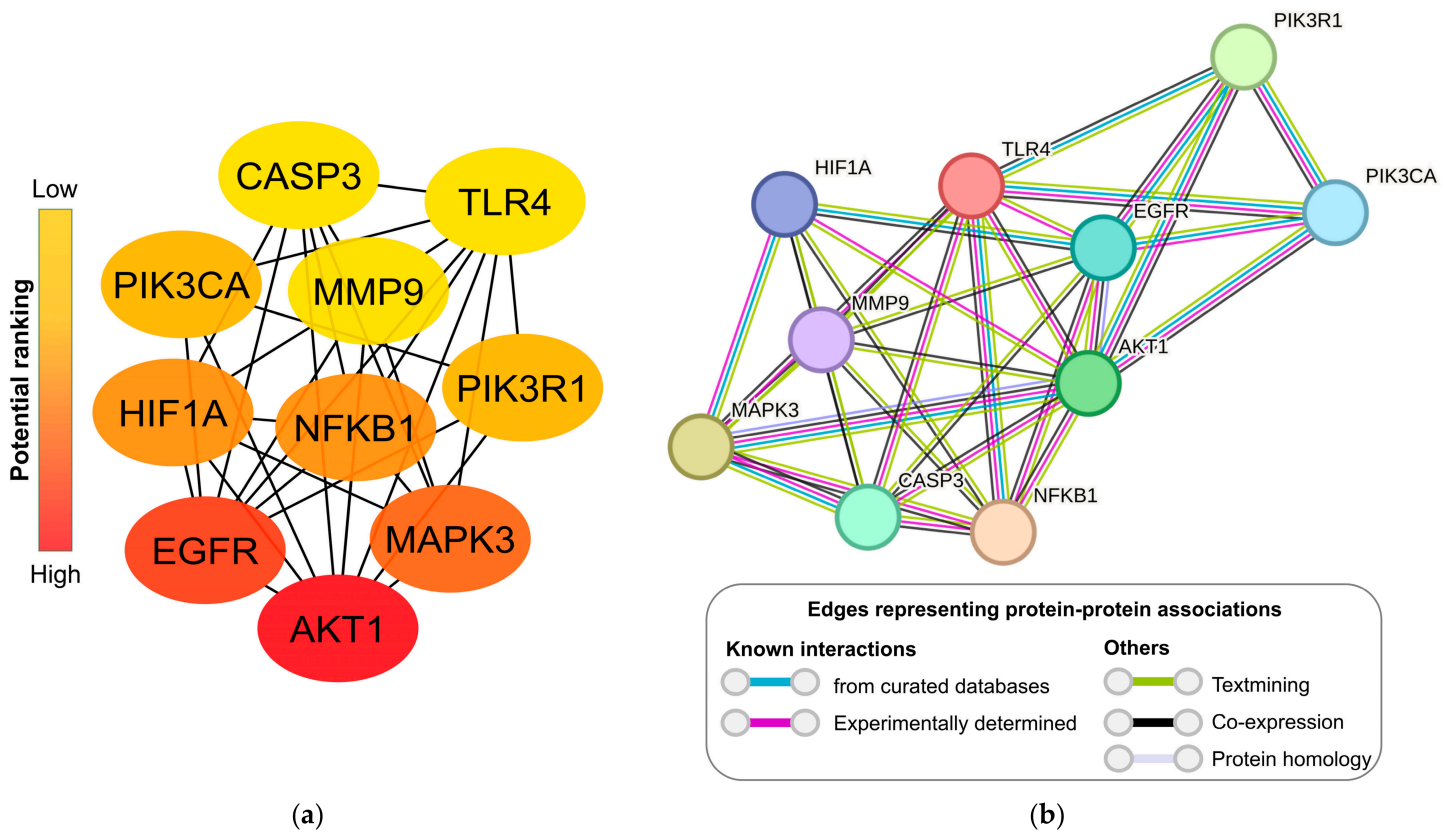
(**a**) The ten potential wound healing-related targets of MLT and its derivatives in the network ranked by degree method and (**b**) their PPI.

Hub gene analysis using CytoHubba (Figure 3a and Table S1) identified the top ten potential wound healing-related targets of MLT and its derivatives; AKT1, EGFR, MAPK3 (ERK1), HIF1A, NFKB1, PIK3CA, PIK3R1, CASP3, MMP9, and TLR4, ranked according to degree interaction. PPI analysis of these ten proteins showed a closely connected network containing 10 nodes and 33 edges, with an average node degree of 6.6, an average local clustering coefficient of 0.86, an expected number of edges at 7, and a PPI enrichment *p*-value less than 1.49 × 10^−13^ (Figure 3b). The interaction network is presented in Table S1.

### 2.3. Function and Pathway Enrichment Analysis of MLT and Derivatives on Wound Healing

**Figure 4:**
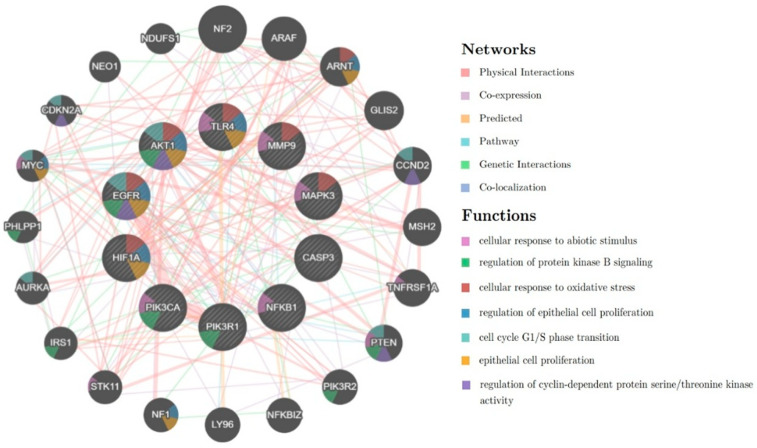
GeneMANIA-based functional enrichment network of the top ten wound healing-related targets of melatonin (MLT) and its derivatives. Inner nodes represent query genes (core targets), and outer nodes represent functionally related genes identified by the database.

The ten potential target proteins (AKT1, EGFR, MAPK3, HIF1A, NFKB1, PIK3CA, PIK3R1, CASP3, MMP9, and TLR4) were analyzed for functional enrichment associated with the different phases of wound healing, including hemostasis, inflammation, proliferation, and remodeling. GeneMANIA (version 3.6.0) analysis generated a functional interaction network (Figure 4) consisting of physical interactions (red), co-expression (purple), predicted interactions (yellow), pathway interactions (light blue), genetic interactions (green), and co-localization (deep blue), accounting for 81.66%, 11.30%, 4.72%, 1.84%, 0.30%, and 0.18% of the network, respectively.

Physical interactions were derived from protein–protein interaction data obtained from BioGRID and PathwayCommons databases [18]. Co-expression relationships were based on correlated gene expression profiles obtained from the Gene Expression Omnibus (GEO) database. Predicted interactions represent potential functional relationships inferred from orthology data. Pathway interactions indicate relationships between genes participating in the same biological pathways and were obtained from Reactome, BioCyc, and PathwayCommons. Genetic interactions were obtained from BioGRID and represent functional associations between genes, and co-localization indicates genes expressed in the same tissue or proteins found in the same cellular compartment.

GeneMANIA identified seven functional categories associated with the input proteins [19]. The enriched functions associated included cellular response to abiotic stimulus, regulation of protein kinase B (AKT) signaling, cellular response to oxidative stress, regulation of epithelial cell proliferation, cell cycle G1/S phase transition, epithelial cell proliferation, and regulation of cyclin-dependent protein kinase activity as presented in Table 1. The seven enriched functions showed false discovery rate (FDR) values ranging from 4.27 × 10^−7^ to 1.58 × 10^−4^. These values were calculated using gene ontology (GO) annotations and are reported as Q-values derived from an FDR-corrected hypergeometric test using the Benjamini–Hochberg algorithm. Coverage ratios, representing the number of annotated genes in the displayed network relative to the total number of genes with the same annotation in the genome, are also reported [19].

According to Table 1, these seven functions can be classified into the inflammation, proliferation and remodeling phases of wound healing [20]. Cellular response to abiotic stimulus and cellular response to oxidative stress are primarily associated with the inflammatory phase. Oxidative stress and excessive ROS production have been reported to contribute to impaired wound healing [4,5,6,20,21,22]. Abiotic stimuli include non-living environmental stressors such as chemicals, pH, temperature, moisture, pollution, and UV radiation, all of which may increase ROS production and oxidative stress within cells [23,24,25]. Excessive ROS can damage dermal cells, whereas lower ROS levels have been associated with improved wound healing [22]. Elevated levels of pro-inflammatory cytokines have also been associated with delayed wound healing [5,6,21].

**Figure 5:**
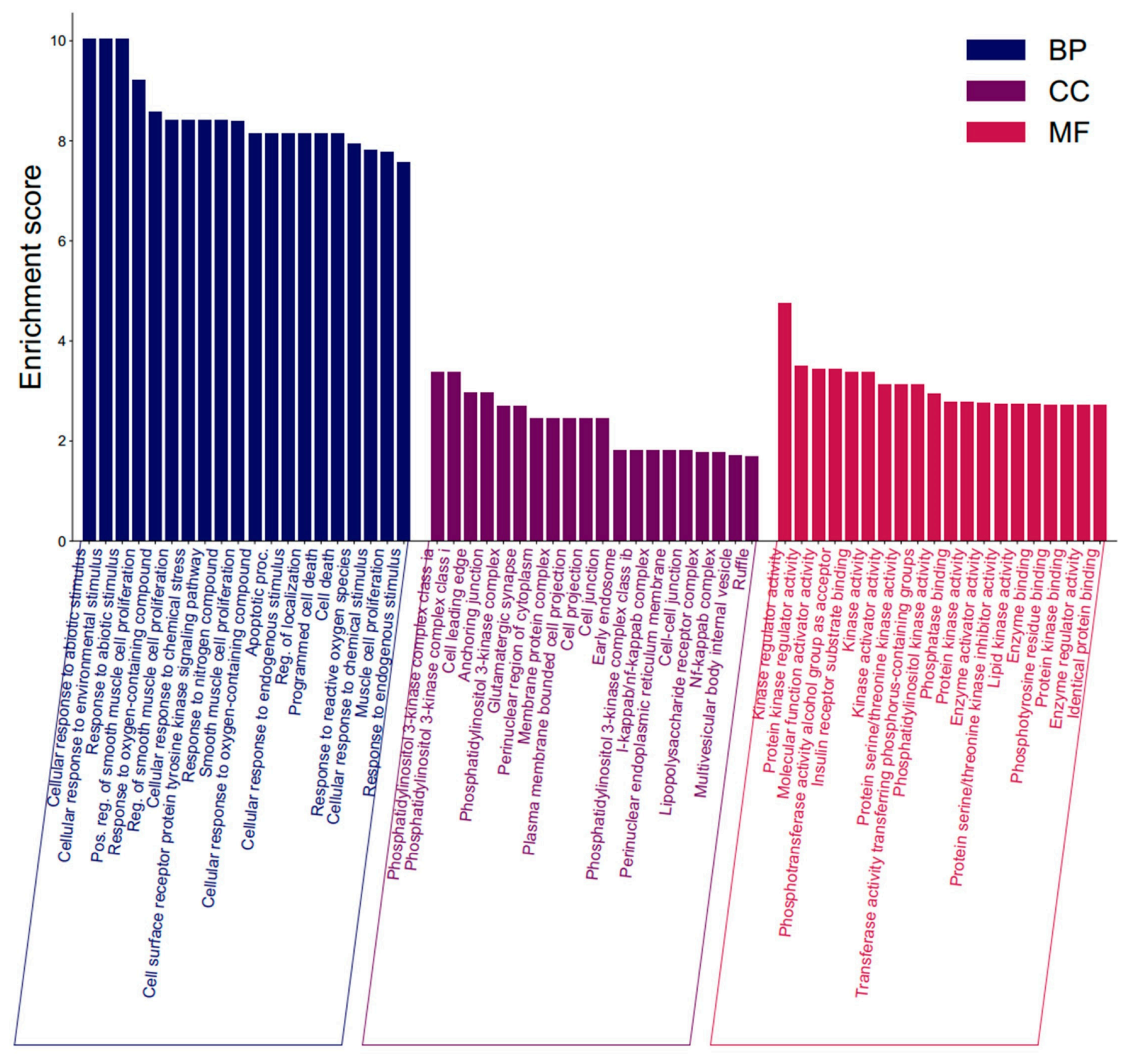
GO enrichments of ten potential proteins are associated with wound healing of MLT and its derivatives; BP: biological process; CC: cellular component; MF: mechanism function.

Another enriched function identified in this study was regulation of protein kinase B (AKT) signaling. Activation of AKT regulates downstream targets involved in cell proliferation, differentiation, apoptosis, and migration, partly through phosphorylation of apoptosis-related proteins such as Bad and caspase-9. AKT signaling also interacts with the NF-κB signaling pathway during the inflammatory phase of wound healing [20]. Previous studies have suggested that activation of the PI3K/AKT signaling pathway promotes epithelial–mesenchymal transition (EMT), which promotes dermal cell healing [26]. Gene ontology (GO) enrichment analysis was subsequently performed to identify enriched biological processes (BP), cellular components (CC), and molecular functions (MF), as shown in Figure 5.

The FDR values for BP, CC, MF, and KEGG enrichment analyses were all below 0.05 (Tables S2–S4), indicating statistically significant enrichment [27]. BP analysis (Figure 5 and Table S2) identified processes associated with the inflammatory phase, including responses to nitrogen compounds, reactive oxygen species, and chemical stimuli, all of which are related to oxidative stress and inflammatory responses [4,5,6,20,21,22,23]. BP analysis also identified positive regulation of smooth muscle cell proliferation, which is associated with angiogenesis, re-epithelialization, and granulation during the proliferative phase [4,5,6]. These findings from SinyGO correlated with the functional enrichment results from GeneMANIA and provide a basis for further in vitro and in vivo studies.

Cellular component (CC) analysis (Table S3) identified enrichment in phosphatidylinositol 3-kinase (PI3K) complex class IA and class I. Cellular components associated with inflammatory signaling, including the I-κB /NF-κB complex, lipopolysaccharide receptor complex, and NF-κB complex, were also identified. Additional enriched cellular response components included membrane protein complexes, plasma membrane-bounded cell projections, cell projections, and cell junctions. MF analysis (Table S4) primarily identified kinase-related regulatory functions [6,20,28].

**Figure 6:**
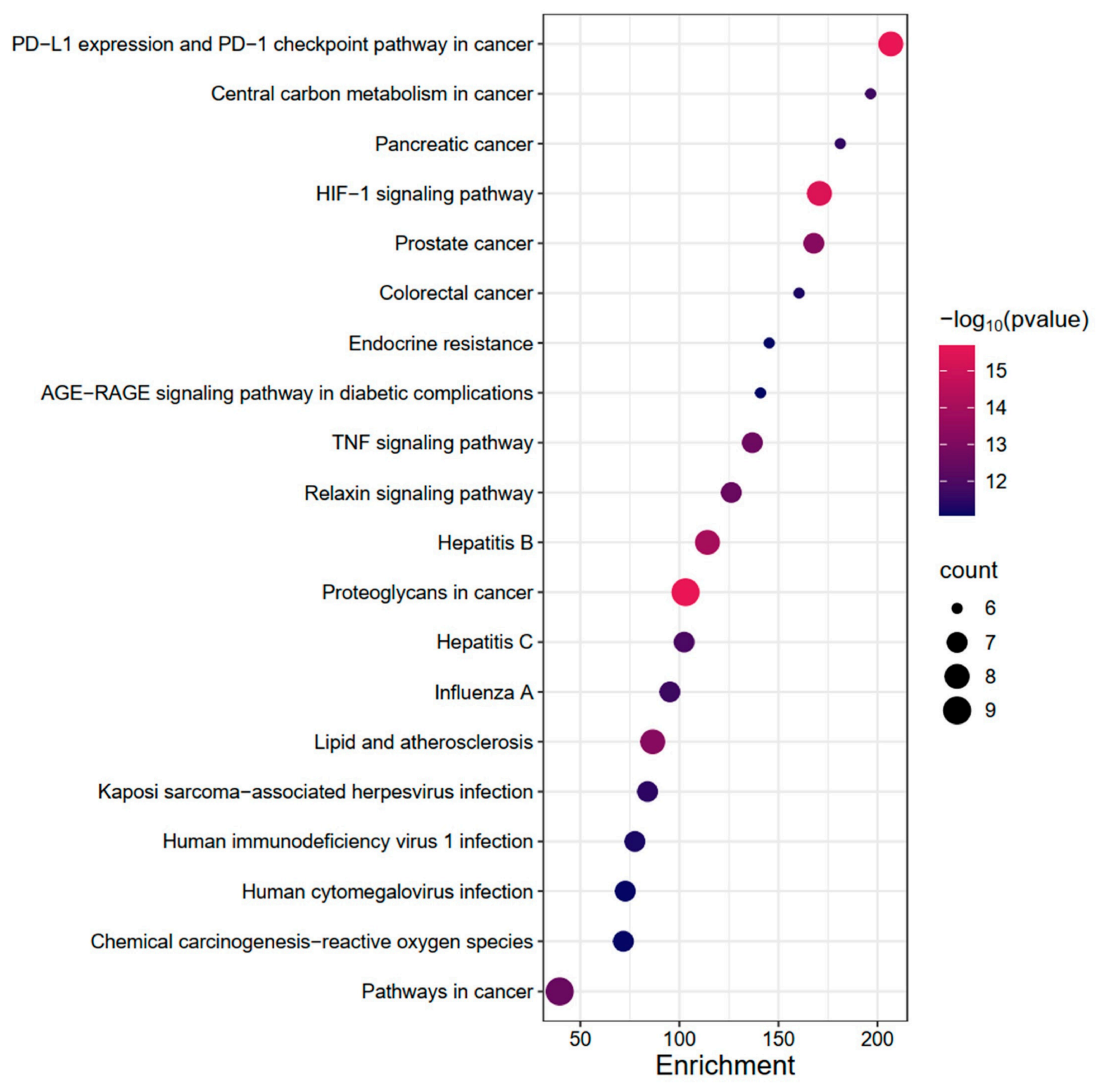
KEGG enrichments of ten potential proteins are associated with wound healing of MLT and its derivatives.

KEGG pathway enrichment identified 20 pathways associated with the ten target proteins (Figure 6 and Table S5). Among these, HIF-1 signaling was highlighted as a candidate regulatory pathway linked to the predicted targets of melatonin derivatives, showing the strongest enrichment score (FDR = 4.48 × 10^−16^) and encompassing eight of the ten target proteins: EGFR, AKT1, HIF1A, NFKB1, PIK3CA, PIK3R1, MAPK3, and TLR4 (Table S6).

**Figure 7:**
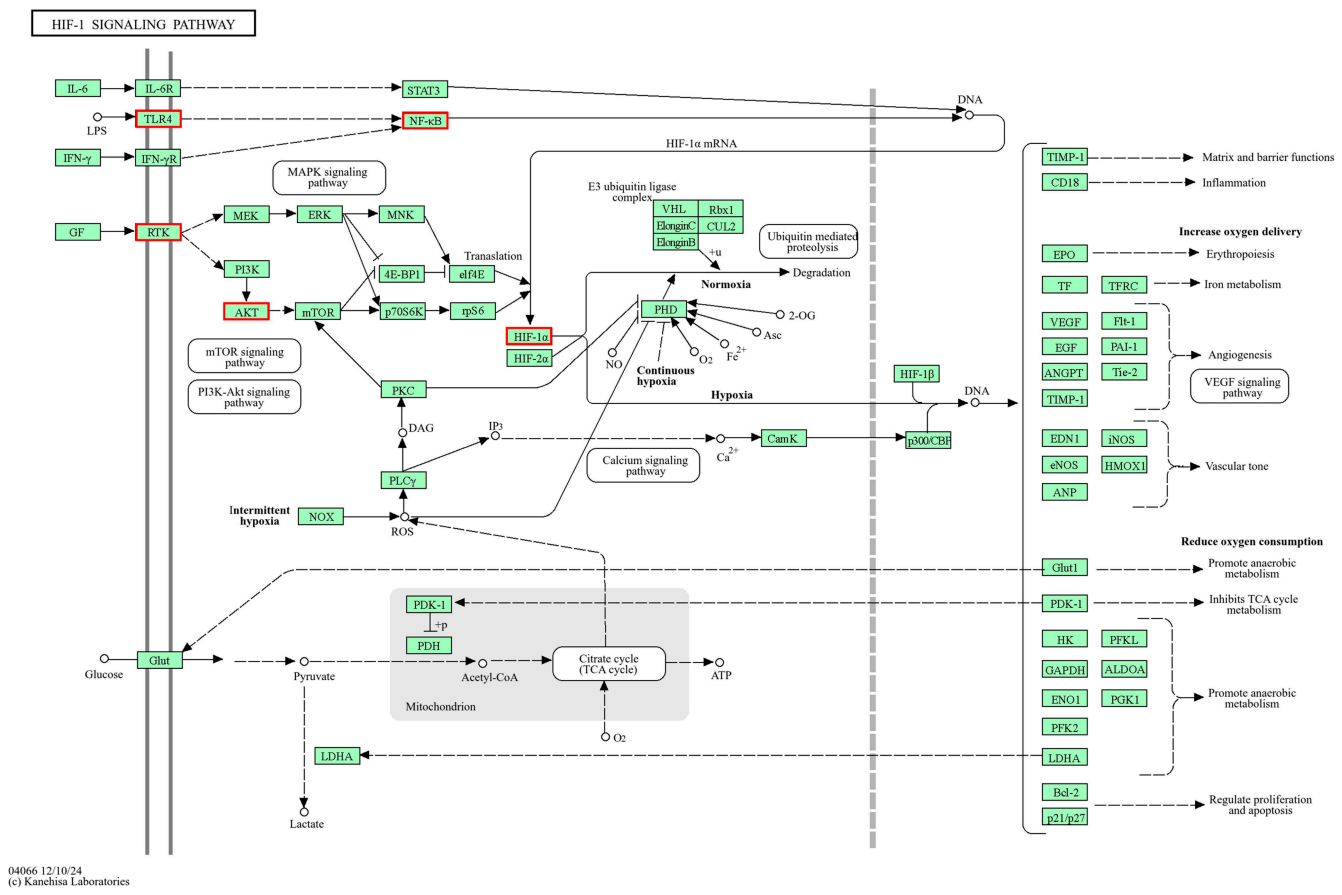
The HIF-1 signaling pathway was retrieved from the KEGG database. Related proteins from the ten potential wound-healing targets of melatonin (MLT) and its derivatives are highlighted in red. The gray area denotes the biological mechanism occurring in mitochondria. Solid arrows indicate activation, dashed arrows indicate indirect effects, and T-shaped arrows indicate inhibition.

The HIF-1 signaling pathway is involved in multiple stages of wound healing, including cell migration, cell survival under hypoxic conditions, cell proliferation, growth factor production, and extracellular matrix (ECM) synthesis [29]. A visualization of the HIF-1 signaling pathway is displayed in Figure 7.

### 2.4. Compounds–Targets–Function–Pathway–Phase of MLT and Its Derivatives on Wound Healing

**Figure 8:**
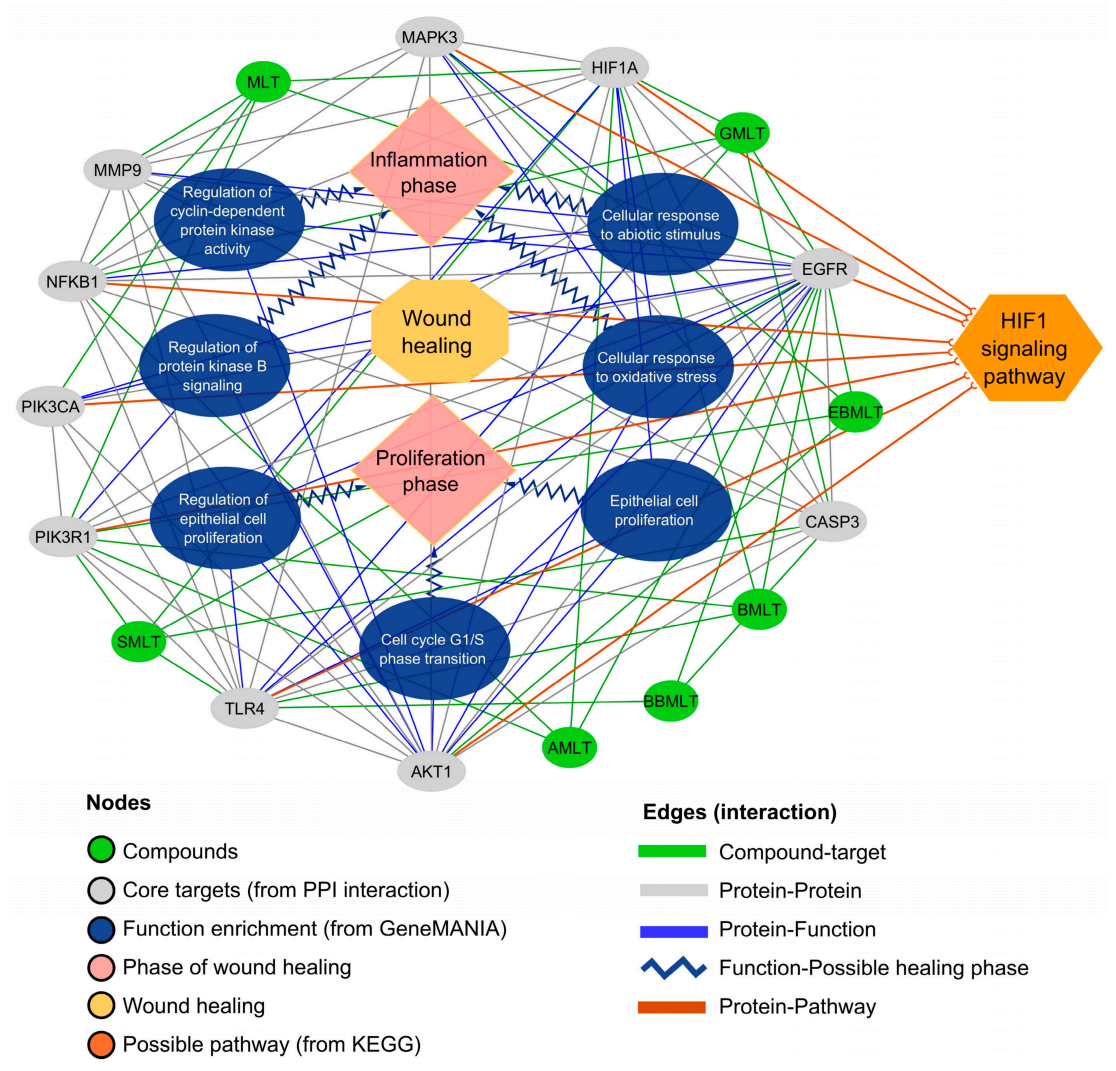
Compounds–targets–function–pathway–phase network of MLT and its derivatives on wound healing-related targets.

Based on our network pharmacology analysis, we constructed a comprehensive compound–target–function–pathway–phase network mapping MLT, its six derivatives, and their associated wound-healing pathways (Figure 8). Topological analysis revealed that the nodes for MLT and GMLT contained six edges; SMLT contained five edges; AMLT, BMLT, and EBMLT contained four edges; and BBMLT contained three edges (Table S7). Their shared intersection targets, including EGFR, AKT1, HIF1A, NFKB1, PIK3CA, PIK3R1, MAPK3, and TLR4, are predicted to play critical roles in the HIF-1 signaling pathway (Figure 7).

During physiological wound healing, HIF-1 signaling is typically activated in response to microenvironmental hypoxia, a condition that predominantly occurs during the inflammatory and early proliferative phases [30,31]. Interestingly, functional enrichment analyses using GeneMANIA (Table 1) and gene ontology (GO) (Figure 5) identified potential biological functions related to cellular responses to abiotic stimuli and oxidative stress, which represent key processes regulated by the HIF-1 pathway during the early inflammatory phase of healing [30]. Furthermore, the literature suggests that the HIF-1 pathway is implicated in angiogenesis, potentially cross-talking with the PI3K/AKT1 pathway (Figure 6) to modulate pro-angiogenic factors such as VEGF, angiopoietin-2, SDF-1, NOS, and adrenomedullin [30]. VEGF is widely recognized as a primary driver of angiogenesis that enhances endothelial cell proliferation, migration, and capillary sprouting. These reported mechanisms align with our GeneMANIA functional predictions, which highlighted the regulation of protein kinase B (AKT) signaling and epithelial cell proliferation (Table 1).

Hypoxia-induced HIF-1 activation has been widely implicated in the literature as a therapeutic target for resolving chronic wounds. Under hypoxic conditions, HIF-1 activation typically supports pro-inflammatory macrophage functions by maintaining ATP production, which in turn preserves cellular motility, invasiveness, and bactericidal activity. Conversely, impaired HIF-1 signaling under hyperglycemic conditions is widely associated with deficient angiogenesis and non-healing diabetic ulcers [7,20,30].

In addition to inflammation and angiogenesis, the HIF-1 pathway is theoretically associated with dermal remodeling. During the remodeling phase, fibroblasts differentiate into myofibroblasts, which synthesize collagen types I and III to rebuild the extracellular matrix [5,31]. However, an excessive or dysregulated fibrogenic response can lead to pathological scarring, such as hypertrophic scars and keloids. Keloid and scleroderma tissues have been reported to exhibit elevated levels of HIF-1α protein relative to normal skin, a phenomenon linked to increased transcription and stabilization of the HIF-1α subunit, which subsequently promotes profibrotic factors like TGF-β1, thrombospondin-1, PAI-1, and VEGF [31]. Consequently, therapeutic modulation of HIF-1 signaling offers a multifunctional approach to balance inflammation, proliferation, and remodeling. Taken together, based on our network pharmacology predictions, among the enriched pathways, HIF-1-related signaling emerged as a candidate regulatory axis associated with the predicted targets of melatonin derivatives. 

### 2.5. Differentially Expressed Genes (DEGs)

To identify datasets reporting the expression levels of wound healing-related targets, the GSE268639 dataset was used for bioinformatics analysis. The GSE268639 dataset includes a total of 318 patients with 24 different types of non-healing wounds and was designed to compare trauma/surgery wounds and mixed arterial/venous leg ulcers with healing wounds [30]. The pooling of ulcers and trauma wounds was intentionally performed to identify a shared core signature of impaired wound healing pathways, rather than disease-specific markers. Under these physiological conditions, both chronic ulcers and severe trauma share common microenvironmental features, such as persistent hypoxia, prolonged inflammation, and impaired extracellular matrix remodeling [30]. Therefore, the GSE268639 dataset was selected to clarify the common shared gene expressions of non-healing wounds.

**Figure 9:**
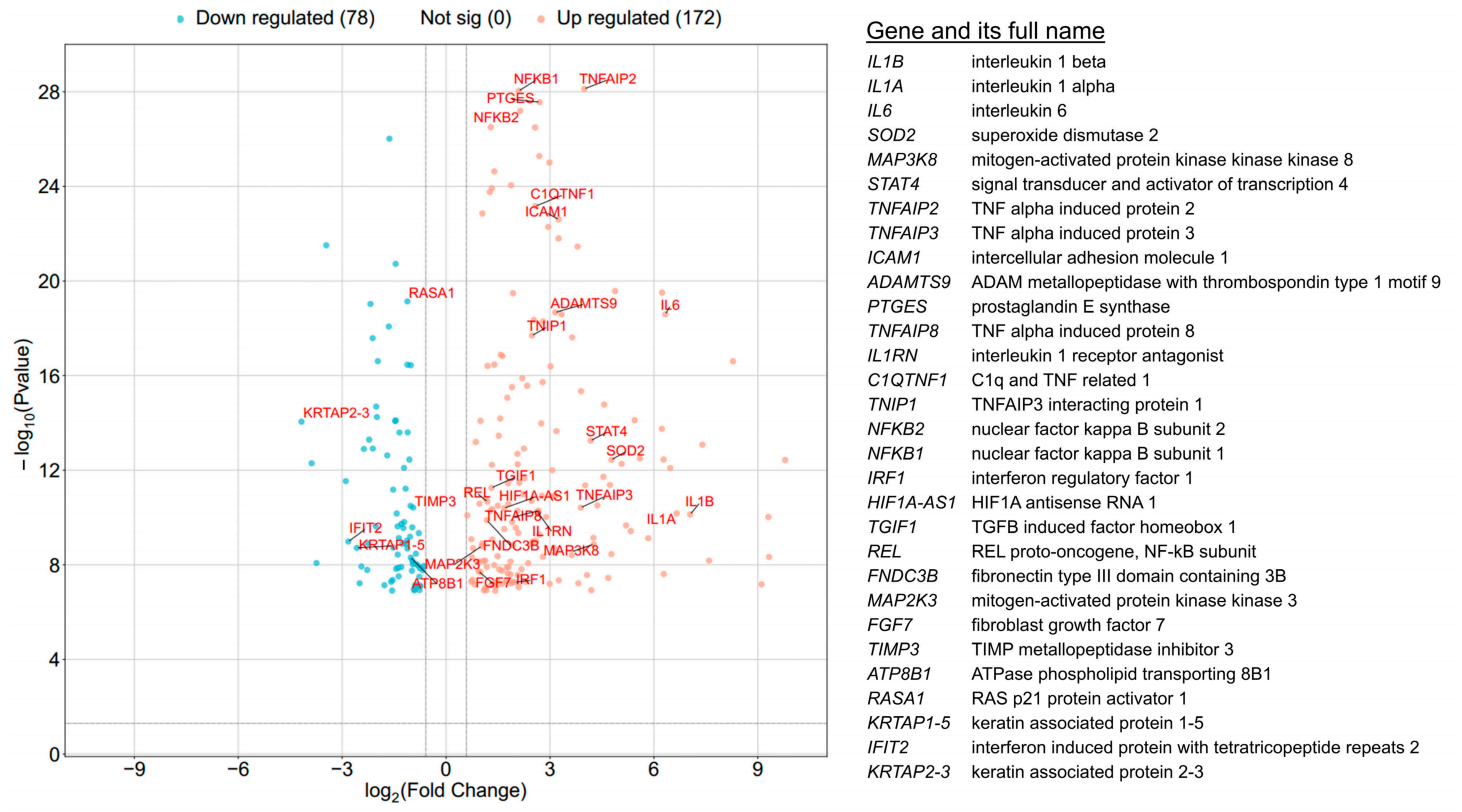
DEGs of non-healing wounds (GSE268639) [30]; blue dots indicate downregulated genes; red dots indicate upregulated genes; the full names of each gene are labeled.

The dataset consisted of three sample groups: healing wound exudates (control), non-healing wound exudates from mixed arterial/venous leg ulcers (non-healing group 1), and non-healing wound exudates from trauma/surgery wounds (non-healing group 2). This dataset was used to analyze differential gene expressions in wound exudates, and the results are presented as a volcano plot in Figure 9. Among the 250 analyzed genes, 172 (red dots) were upregulated and 78 (blue dots) were downregulated in non-healing wounds (Figure 9). The upregulated genes included IL1B, IL1A, IL6, SOD2, MAP3K8, STAT4 TNFAIP2, ICAM1 PTGES, NFKB2, NFKB1, REL, and HIF1A-AS1, which are associated with oxidative stress and inflammatory responses [6,20,21,30]. In addition, ADAMTS9, HIF1A-AS1, and TGIF are associated with re-epithelialization, angiogenesis, and tissue remodeling [6,20,30,32]. In contrast, the downregulated genes included TIMP3, ATP8B1, RASA1, KRTAP1-5, IFIT2, and KRTAP2-3, which are associated with cell proliferation and tissue remodeling [6,30]. The DEGs identified in non-healing wounds were consistent with the network pharmacology analysis, particularly for NFKB1 and HIF1A (Figure 9). These findings suggest that the HIF-1 and NF-κB pathways may represent potential therapeutic targets of MLT and its derivatives in wound healing.

### 2.6. Molecular Interaction of MLT and Derivatives on Key Targets

Our network pharmacology findings indicated the HIF-1 signaling pathway as a candidate pathway associated with the predicted targets of melatonin derivatives. However, we acknowledge that direct experimental validations—such as monitoring HIF-1α protein stabilization, nuclear translocation, or downstream effectors (e.g., VEGF, GLUT1, or PDK1)—were not performed. Thus, the HIF-1 pathway is presented here as a predictive framework that provides a basis for future molecular validation. Based on this study, HIF-1-related signaling was highlighted as a candidate regulatory axis by network enrichment analysis, which played a vital role in different phases of wound healing, including cell migration, survival, proliferation, growth factor production, and extracellular matrix synthesis [31,33]. Many studies have suggested that positive regulation of HIF-1 plays a vital role in wound-healing progression [29,34,35]. Our in silico findings suggest that MLT and its derivatives may influence wound healing by modulating tissue repair mechanisms. Following tissue injury, disruption of blood vessels creates a localized hypoxic environment, which serves as a key trigger for the wound-healing cascade. HIF-1 functions as a central signaling hub by integrating signals from inflammatory mediators such as NF-κB, growth factor receptors including EGFR and VEGFR, and ECM remodeling enzymes like MMP-1 and MMP-13.

The protein–ligand complexes were validated by re-docking the co-crystallized ligand into each target protein, followed by analysis of the root mean square deviation (RMSD). The RMSD values for most targets were within the accepted range (RMSD ≤ 2.0), whereas MMP-1 exhibited an acceptable value (2.0 < RMSD < 3.0) [36], as shown in Table 2. Superposition of the original and re-docked ligands for all selected proteins is presented in Figure S1.

**Figure 10:**
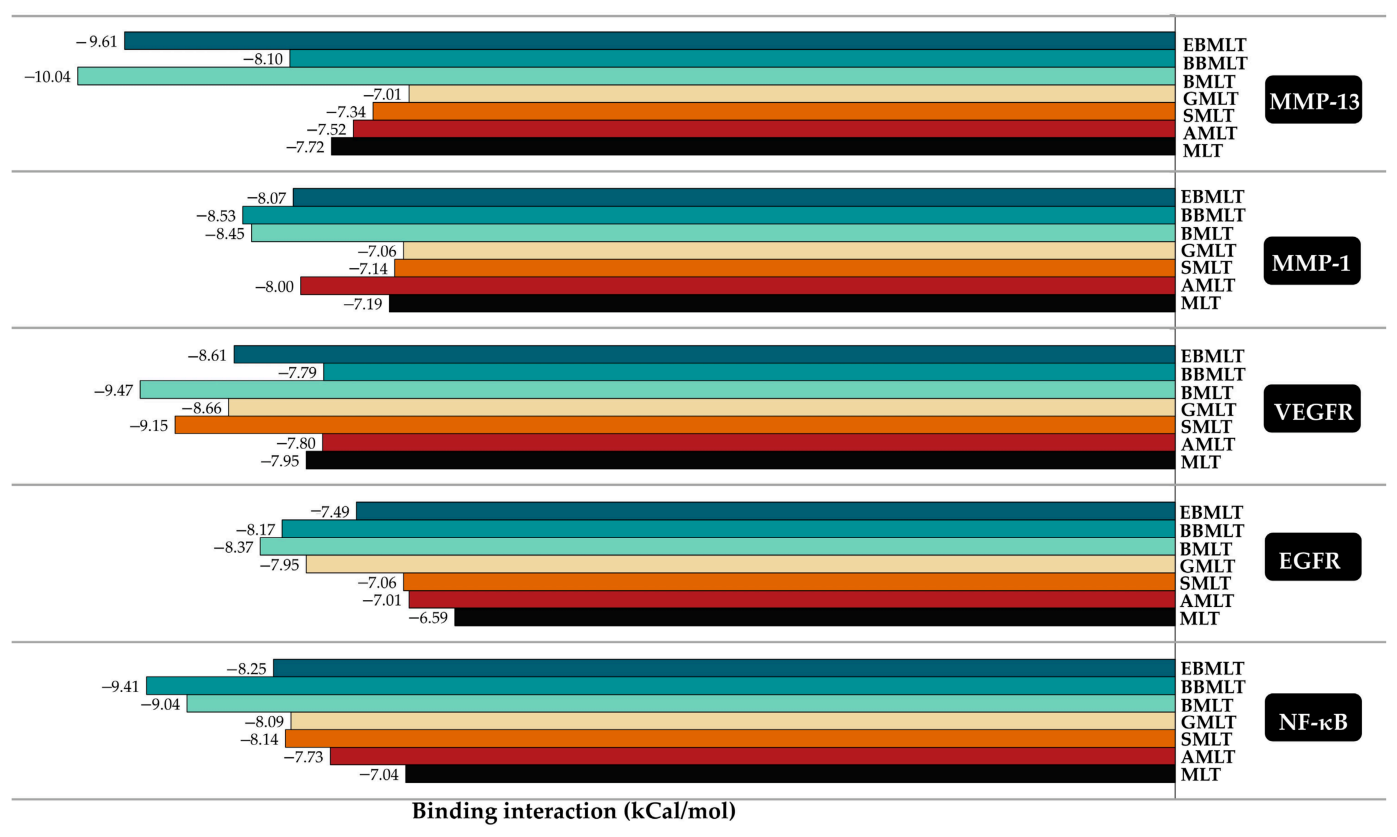
Binding affinity of MLT and six derivatives with NF-κB, EGFR, VEGFR, MMP-1, and MMP-13.

As shown in Figure 10, most compounds were predicted to have favorable binding energies toward all target proteins, with binding energies below −7.0 kcal/mol [37,38]. The only exception was the MLT-EGFR complex (−6.59 kcal/mol). Compared with the MLT parent compound, all six derivatives were predicted to have more favorable binding interactions across all target proteins, suggesting that MLT derivatives at the *N1*- or *N2*-positions may exert potential wound-healing effects through predicted interactions with key targets across multiple phases of the healing cascade.

The inflammatory phase is the first active stage of wound healing and is regulated by several inflammatory signaling pathways. NF-κB not only regulates the production of pro-inflammatory cytokines but also acts as a transcriptional activator of the HIF-1α gene [31,35]. Cross-talk between NF-κB and HIF-1α links inflammatory signaling with the cellular response to hypoxia. This interaction may prime injured tissues by increasing HIF-1A expression before severe hypoxia develops, which may support the progression from the inflammatory phase to the proliferative phase of wound healing. Therefore, NF-κB was selected for molecular docking to evaluate the potential interactions of MLT and its derivatives with an important inflammatory target.

**Figure 11:**
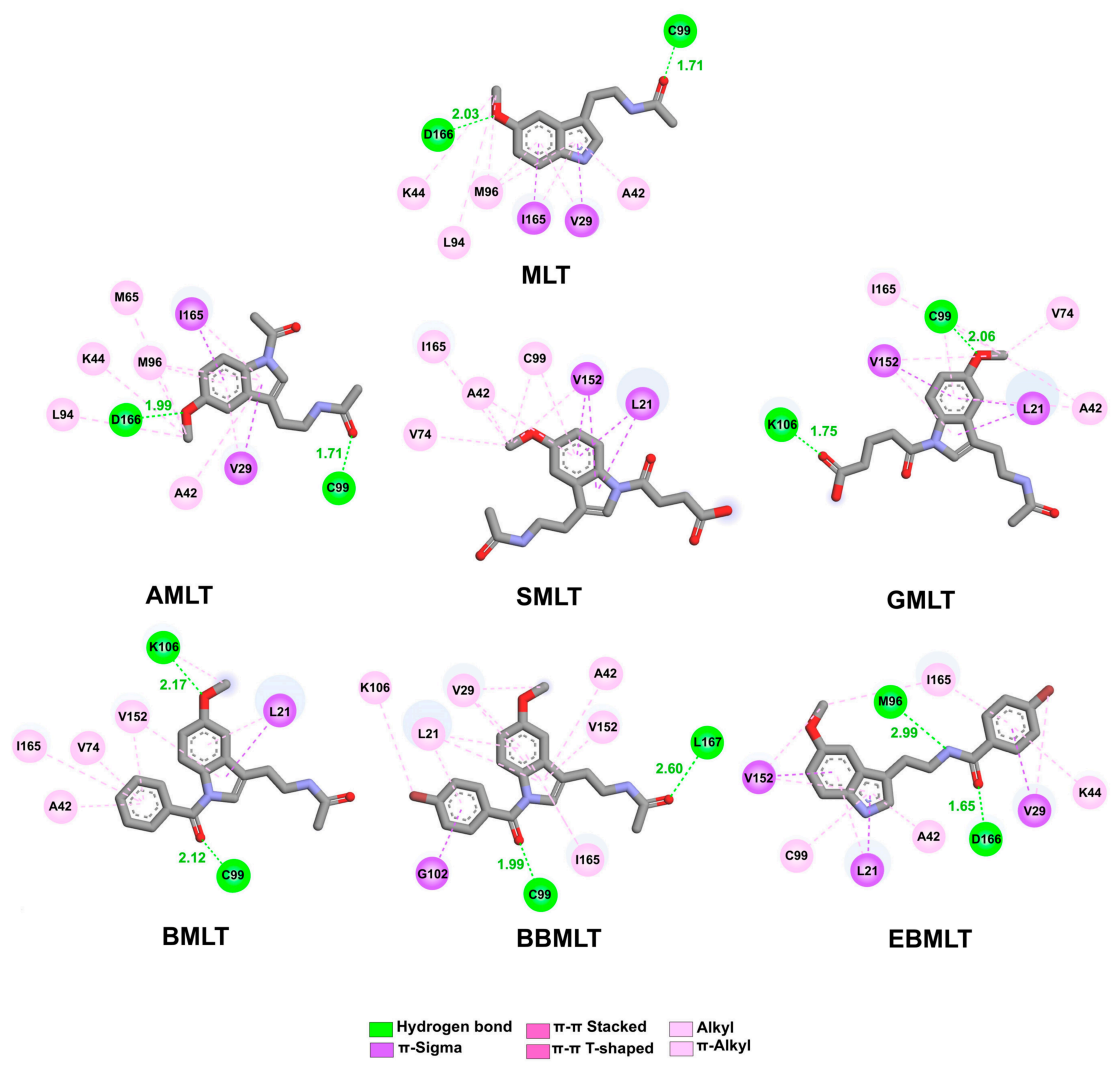
The binding interactions of MLT and its derivatives with the NF-κB protein.

According to the molecular docking results, BBMLT, BMLT, and EBMLT exhibited potential interaction mode with NF-κB (Figure 10). BBMLT formed two hydrogen bonds with residues C99 (1.99 Å) and L167 (2.60 Å) (Figure 11 and Table S8). These hydrogen bond interactions may contribute to the strong binding affinity of BBMLT toward NF-κB. BBMLT also formed seven hydrophobic interactions, including five key residues (L21, V29, A42, V152, and I165) that have been reported to be important for inhibitor bindings [39].

**Figure 12:**
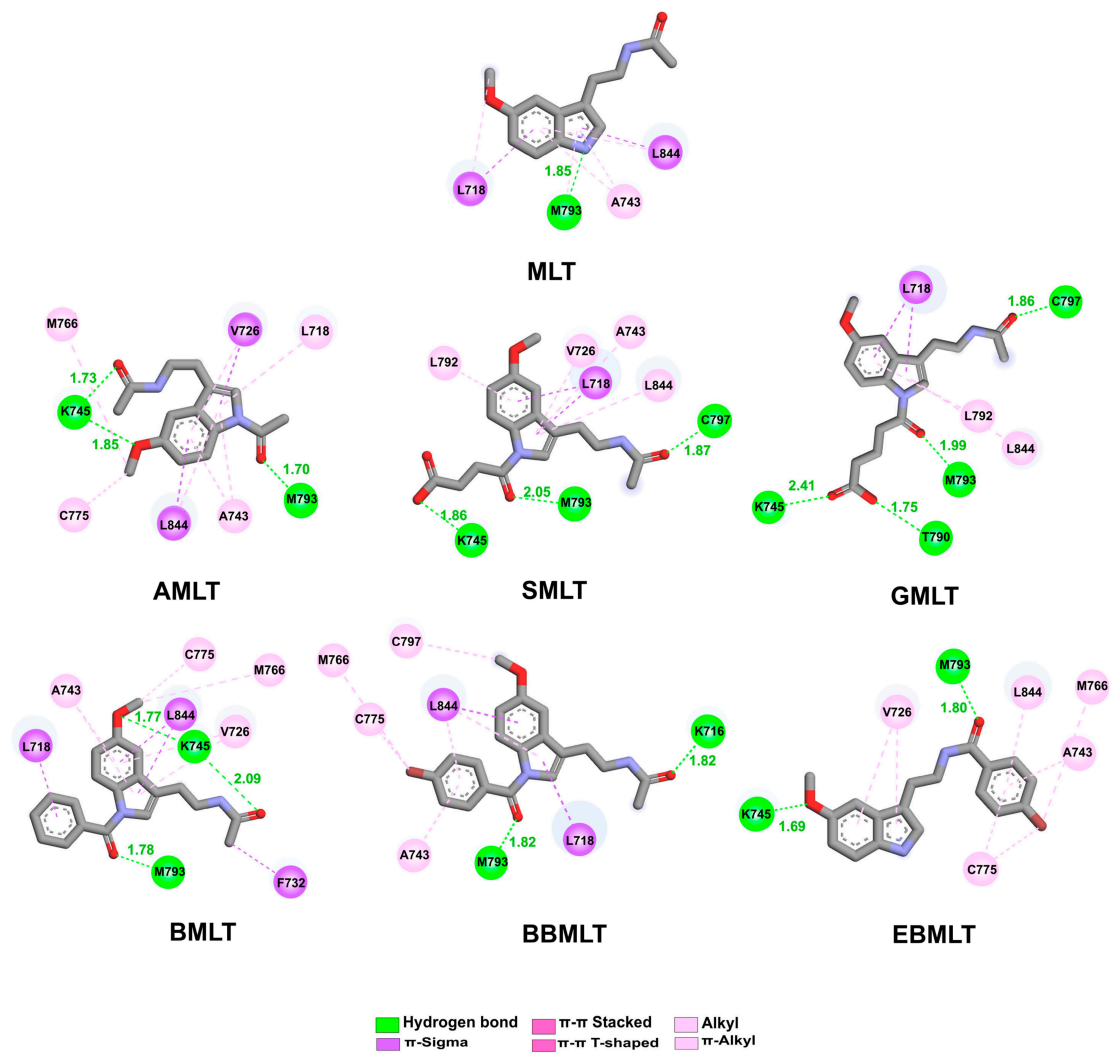
The binding interactions of MLT and its derivatives on the EGFR protein.

The proliferative phase requires rapid migration and proliferation of keratinocytes to close the wound, a process mediated by EGFR signaling. Activation of EGFR via the PI3K/Akt pathway has been shown to stabilize HIF-1α protein, even under near-normal oxygen levels [32]. Conversely, HIF-1 has been reported to influence the expression of EGFR ligands, thereby supporting keratinocyte migration [40]. Molecular docking predicted that all derivatives would form a hydrogen bond with M793, similar to the FDA-approved drug afatinib [41]. In addition, all derivatives were predicted to form a hydrogen bond with K745, which represents a critical interaction pose often associated with known EGFR inhibitors [42] (Figure 10). Among all compounds, BMLT, BBMLT, and EBMLT were predicted to have favorable binding interactions for EGFR (Figure 10). As shown in Figure 12 and Table S9, these three compounds were also predicted to form hydrophobic interactions, especially with BMLT forming seven predicted interactions with the EGFR active site. These computational results indicate that aromatic substitution of MLT may improve predicted binding compatibility within the ATP-binding pocket of EGFR compared with aliphatic substitution.

HIF-1α is a well-established transcription factor, known to play a central role in tissue regeneration under hypoxic conditions [6,30,43]. In the oxygen-deprived wound environment, the stabilization of HIF-1α and its subsequent dimerization into the active HIF-1 complex is known to initiate a highly coordinated gene expression program. Rather than driving a simple, single-signal pathway, HIF-1 pathway has been reported to influence both sides of the angiogenic equation: the ligand and the receptor. Specifically, HIF-1α binds to hypoxia response elements, which are associated with increased VEGF secretion. Simultaneously, HIF-1α binds directly to the promoter of the VEGFR-1 gene, which has been associated with the regulation of VEGFR-1 expression on flanking endothelial cells and infiltrating macrophages [6,30,43]. This synchronized upregulation makes VEGFR-1 a highly compelling model for study. Because VEGFR-1 is a direct downstream target of HIF-1α, studying its spatial expression and decoy function provides a useful model for investigating the potential involvement of HIF-1α in modulating the cellular transition from hypoxic inflammation to organized vascular remodeling, rather than chaotic vessel sprouting. This process is essential for delivering the oxygen and nutrients required to sustain the high metabolic demands of regenerating tissue. Thus, VEGFR-1 was selected as a key target to investigate the functional dynamics of the HIF-1 signaling pathway during wound angiogenesis. 

**Figure 13:**
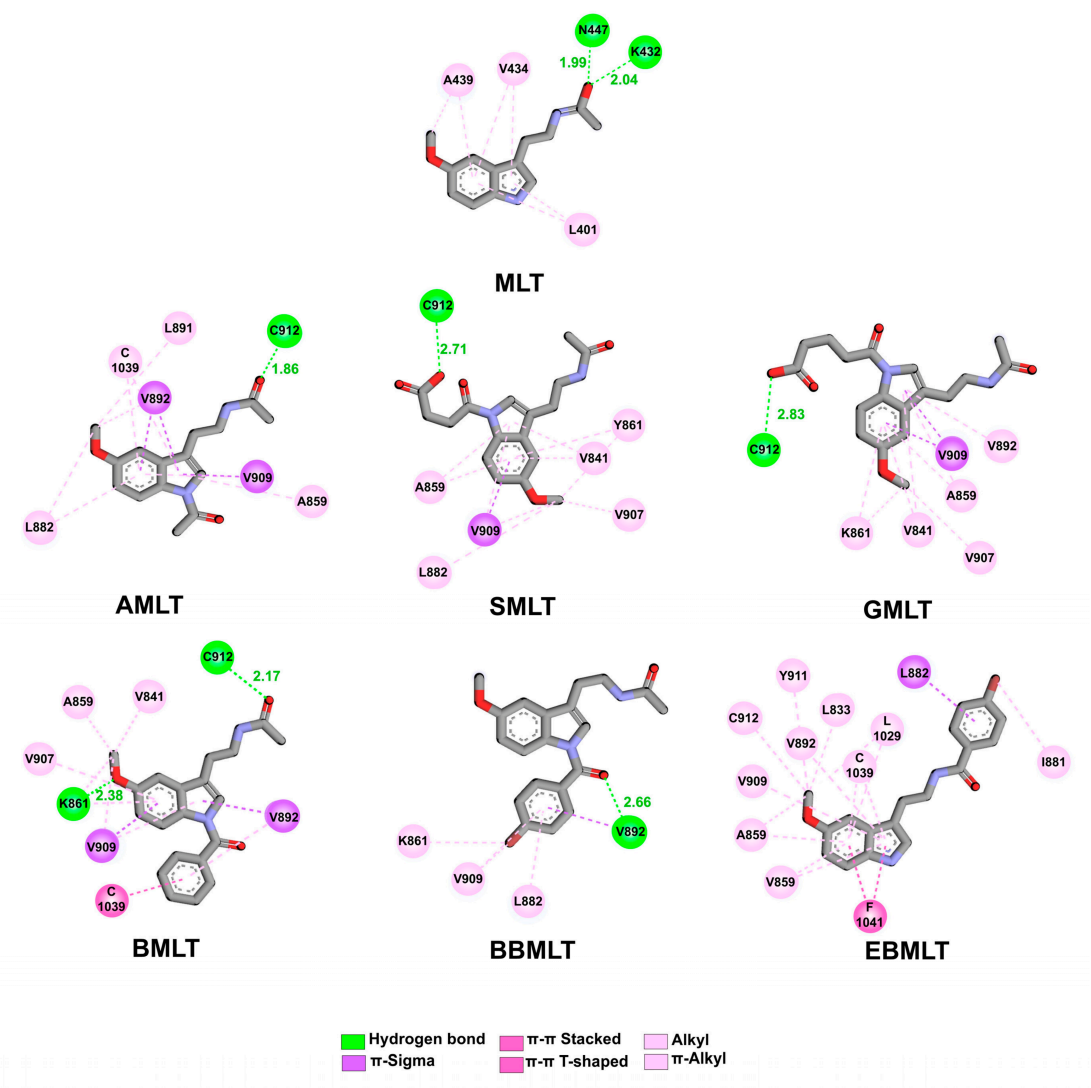
The binding interactions of MLT and its derivatives with the VGFR protein.

Molecular docking of all compounds with VEGFR-1 predicted favorable binding interactions for BMLT, SMLT, and GMLT (−9.70, −9.15, and −8.66 kcal/mol, respectively). All three compounds were predicted to form contacts with amino acid residues 803–911, which comprise the protein kinase domain of the VEGFR family [44]. BMLT adopted a favorable predicted binding position within the VEGFR-1 kinase domain, forming two predicted hydrogen bonds with K861 (2.38 Å) and C912 (2.17 Å) (Figure 13 and Table S10). These predicted interactions are similar to those reported for lenvatinib and 5-fluorouracil (5-FU), which have also been reported to interact with VEGFR through K861 and C912 [45]. Moreover, the *N1*-aliphatic substitution (AMLT, SMLT, and GMLT) with longer side chains appeared to show predicted binding compatibility with VEGFR-1.

**Figure 14:**
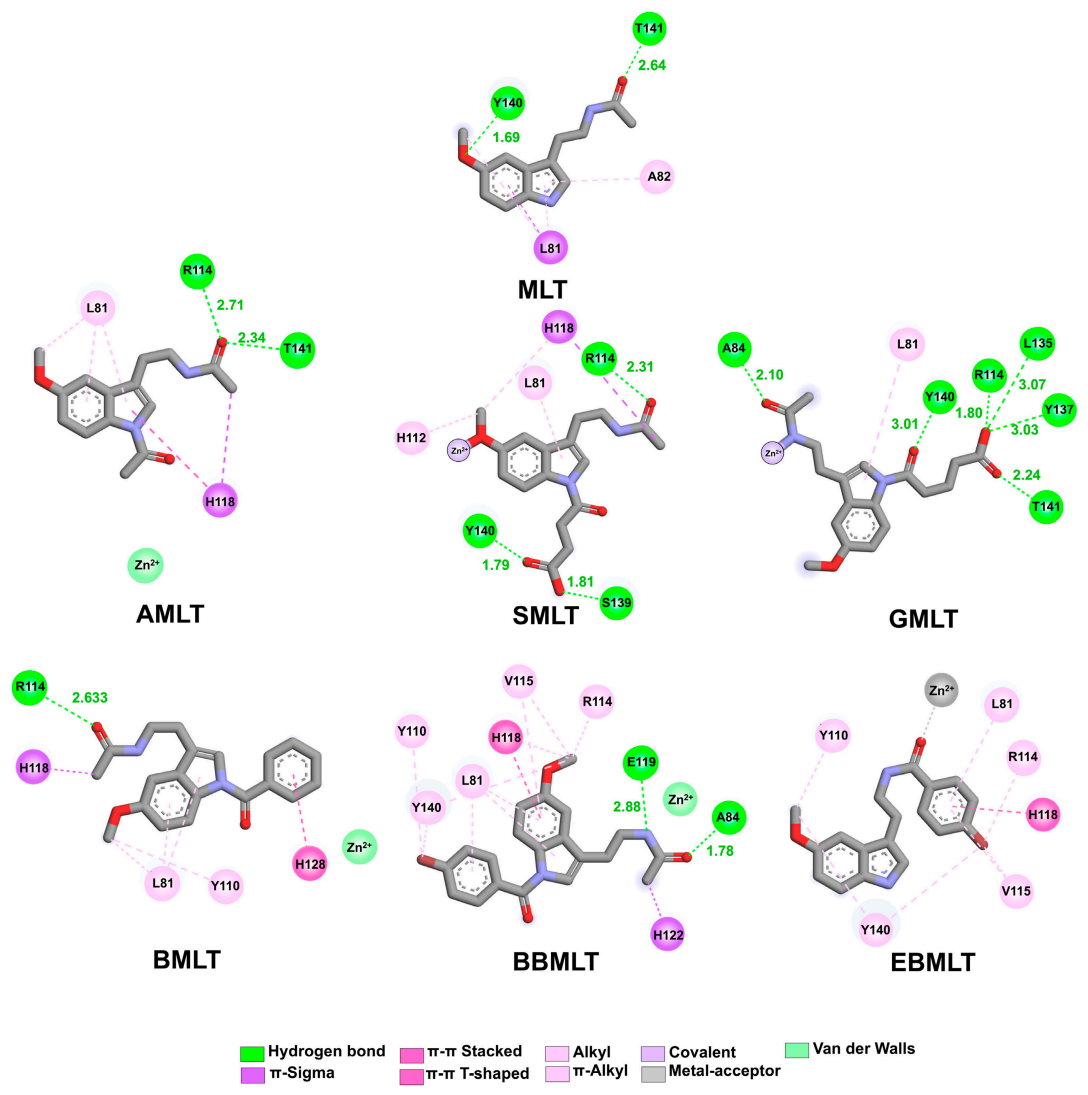
Binding interaction of MLT and six derivatives with MMP-1.

Extracellular matrix (ECM) remodeling, though characterizing the final phase of wound healing, relies on enzymatic dynamics initiated during earlier stages of tissue repair. This crucial remodeling process is primarily mediated by the matrix metalloproteinase (MMP) family [46]. Among these, MMP-1 (collagenase-1) is critical during early re-epithelialization; by degrading Type I collagen, it is associated with keratinocyte detachment and lateral migration across the wound bed [7,47]. Subsequently, MMP-13 (collagenase-3) is involved in collagen maturation, which contributes to granulation tissue remodeling and wound contraction [7,47]. Crucially, both enzymes are transcriptionally upregulated in response to the HIF-1 pathway. This hypoxia-mediated induction of MMPs is important for maintaining the provisional matrix dynamically flexible for cell infiltration while supporting the eventual replacement of temporary granulation tissue by a highly organized, permanent scar [7,47]. Consequently, this phenomenon suggests that HIF-1α functions not only as an initiator of angiogenesis, but also as a potential temporal coordinator of ECM remodeling. Therefore, both MMP-1 and MMP-13 were selected as key targets to study the functional outcomes of the HIF-1 signaling pathway during the remodeling phase of wound healing. Molecular docking with MMP-1 predicted favorable binding interactions for BBMLT and BMLT, followed by EBMLT. These three derivatives were predicted to interact with selected amino acid residues of MMP-1 in the S1’ pocket of MMP-13 [48,49]. The two best-performing derivatives, BBMLT and BMLT, were predicted to have binding energies of −8.53 kcal/mol and −8.45 kcal/mol, respectively (Figure 10). Both derivatives were predicted to form more hydrophobic interactions than other compounds (Figure 14 and Table S11). BBMLT was predicted to form three hydrophobic interactions with MMP-1’s catalytic histidine residues H118 and H122, together with Y140, which is part of the specific loop that surrounds the Zn-distal region of the S1’pocket [48,49].

**Figure 15:**
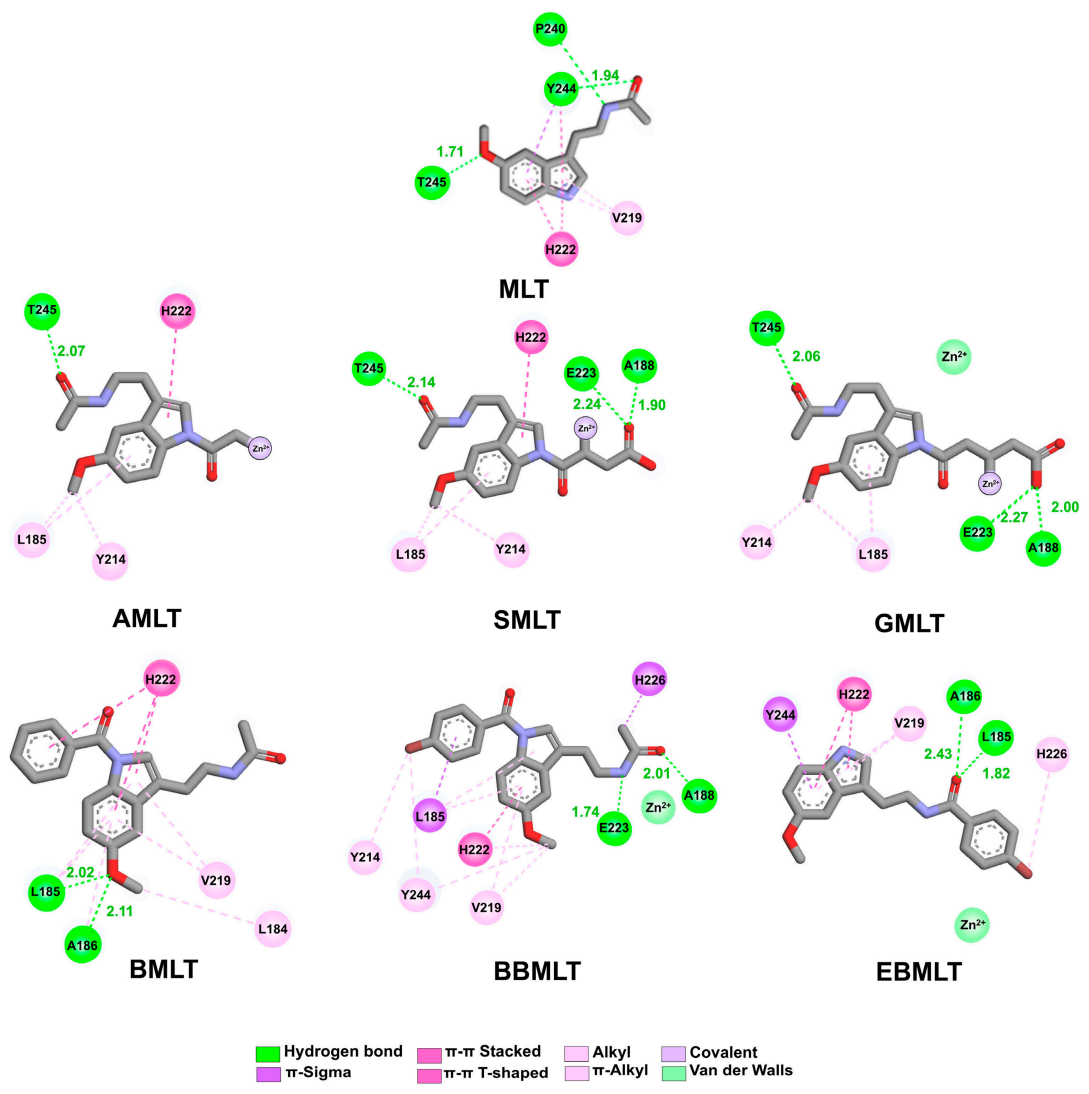
Binding interaction of MLT and six derivatives with MMP-13.

Another important target associated with wound healing is MMP-13. Molecular docking of all compounds with MMP-13 predicted favorable binding interactions for BMLT, EBMLT, and BBMLT. BMLT was predicted to have the most favorable binding interactions for MMP-13 (−10.04 kcal/mol) compared with all compounds (Figure 10). Based on the molecular docking analysis with MMP-13 (Figure 15 and Table S12), BMLT was predicted to form hydrogen bonds with L185 (2.02 Å) and A186 (2.11 Å). These short bond distances indicate a predicted interaction characterized by moderate-to-strong hydrogen-bonding networks [49]. It was also predicted to form five hydrophobic interactions with L184, L185, A186, V219, and H222. The common interactions predicted for BMLT, EBMLT, and BBMLT with MMP-13 were hydrophobic interactions involving H222 and H226 (the catalytic histidine residues) together with the MMP-13 specific loop residue Y244 [48].

Based on these molecular docking simulations, this preliminary computational study suggests that MLT derivatives may possess potential wound-healing properties through predicted interactions with key targets involved in the healing process. The top three ranked ligand–protein interactions were generally observed for MLT derivatives substituted with aromatic groups (benzoyl and bromobenzoyl). An exception was VEGFR-1, which showed more favorable predicted binding interactions for the aliphatic substitutions succinoyl (SMLT) and glutaryl (GMLT) groups, ranked second and third, respectively. Nevertheless, aromatic groups, particularly the benzoyl group, exhibited better overall predicted interactions across the targets.

**Figure 16:**
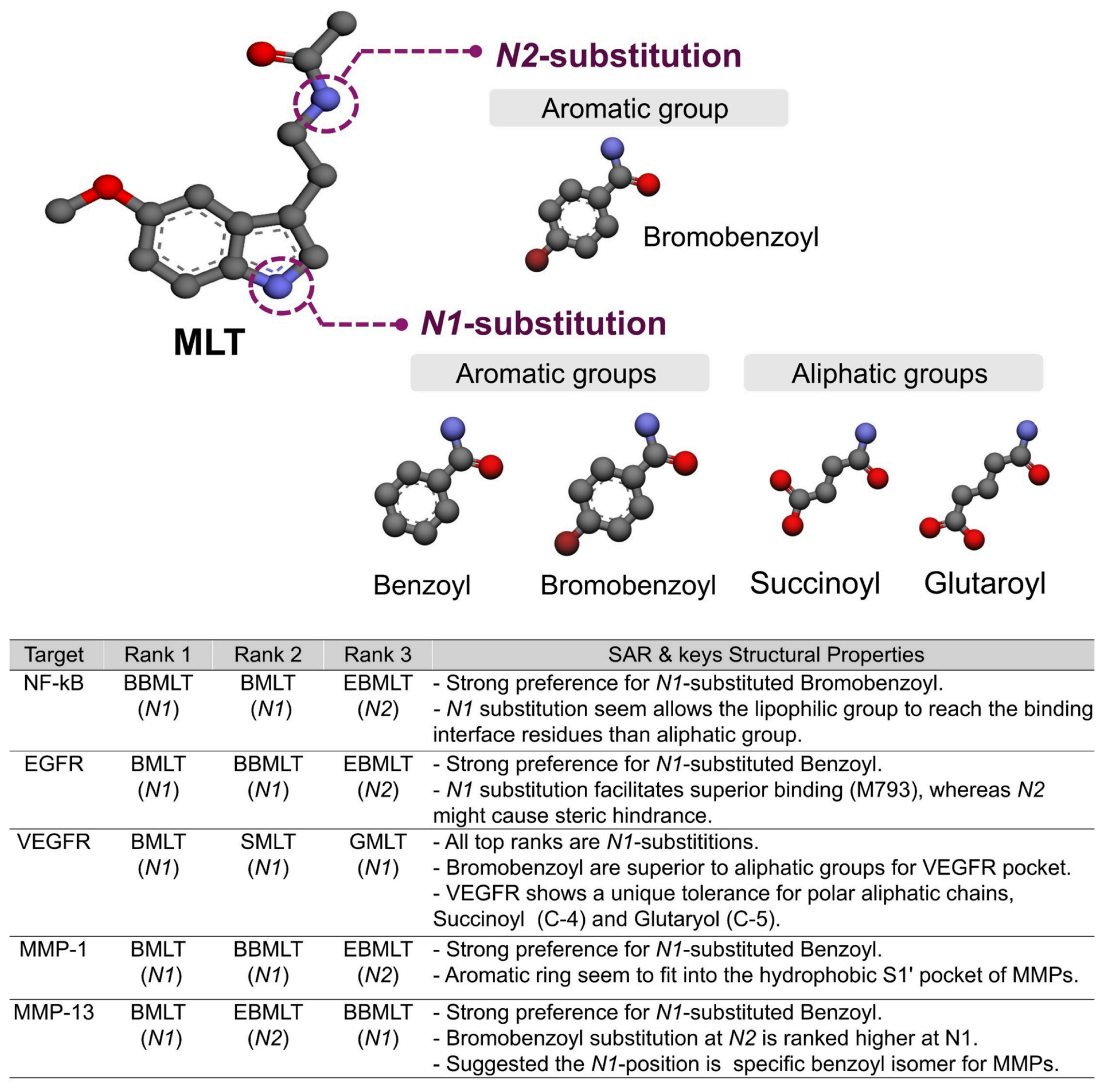
Structure–activity relationships (SAR) of MLT derivatives with key targets on wound healing, NF-κB, EGFR, VEGFR, MMP-1, and MMP-13 based on molecular docking results.

Our molecular docking simulation suggests that structure–activity relationship (SAR) evaluations of predicted wound-healing activity are highly dependent on the modification site; specifically, *N1*-substitution was generally associated with more favorable predicted binding interactions toward NF-κB, EGFR, VEGFR-1, MMP-1, and MMP-13 compared with *N2*-substitution (Figure 16). Molecular modeling suggested that *N1*-functionalization may optimize alignment with critical binding pockets, such as interaction with EGFR residue M793, while *N2*-functionalization may introduce predicted s steric hindrance, which may limit pocket accessibility.

### 2.7. Biological Response of MLT and Its Derivatives on Normal Human Dermal Fibroblasts

In accordance with our network pharmacology predictions, MLT and its six derivatives exert therapeutic effects on wound healing via the HIF-1 signaling pathway, a central cascade modulating inflammation, cell proliferation, and tissue remodeling. These computational insights align with our previously established in vitro and in vivo anti-inflammatory data [10,11,12]. To biologically validate these in silico models, the effects of these compounds on cell proliferation and migration during the proliferative phase of healing were investigated. Normal human dermal fibroblast (NHDF) cells were selected as the in vitro model, given their vital role in wound closure and skin remodeling [6].

**Figure 17:**
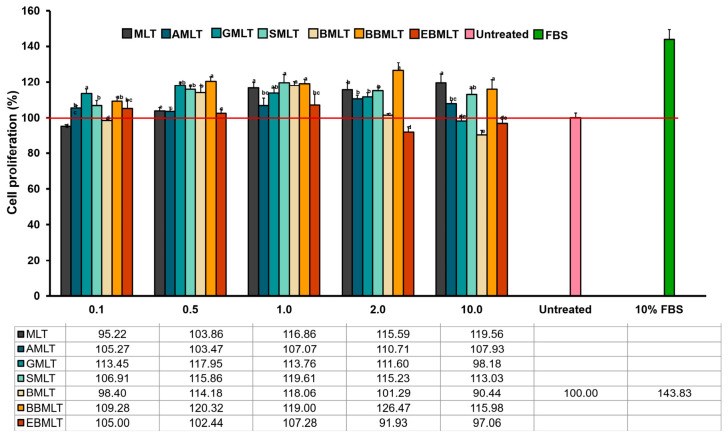
Effect of MLT derivatives on NHDFs proliferation at 24 h. Results were normalized to untreated cells (100%, into red line). Untreated means FBS-free group and 10% FBS means positive control group; data indicated in mean ± SD (*n* = 5). ^a–e^ means significant difference at *p* < 0.05 by Tukey’s HSD.

Following 24 h of incubation across a concentration range (0.1, 0.5, 1.0, 2.0, and 10 µM), all compounds were non-toxic, maintaining NHDF cell viability above 80% [50]. As shown in Figure 17, all tested compounds significantly enhanced NHDF viability compared to the untreated control (FBS-free medium, red line). The parent compound, MLT, significantly enhanced cell viability at concentrations of 1, 2, and 10 µM (*p* < 0.001) over 24 h. Among the derivatives, AMLT induced a highly significant increase in cell viability at 1, 2, and 10 µM (*p* < 0.001), while remaining effective at concentrations as low as 0.1 µM (*p* < 0.001). Prominent effects on cell viability were also observed for the other derivatives across specific ranges: SMLT (0.5–10 µM, *p* < 0.001), GMLT (0.1–2 µM, *p* < 0.001), BMLT (0.5–1.0 µM, *p* < 0.001), BBMLT (0.1 µM, *p* < 0.01; and 0.5–10 µM, *p* < 0.001), and EBMLT (1.0 µM, *p* < 0.05). Notably, at a standardized concentration of 1 µM, MLT and all six derivatives yielded significantly higher viability rates than the untreated group. This robust in vitro response in NHDF cells correlates with our computational predictions, supporting that MLT and its derivatives may enhance NHDF cell viability and activity.

**Figure 18:**
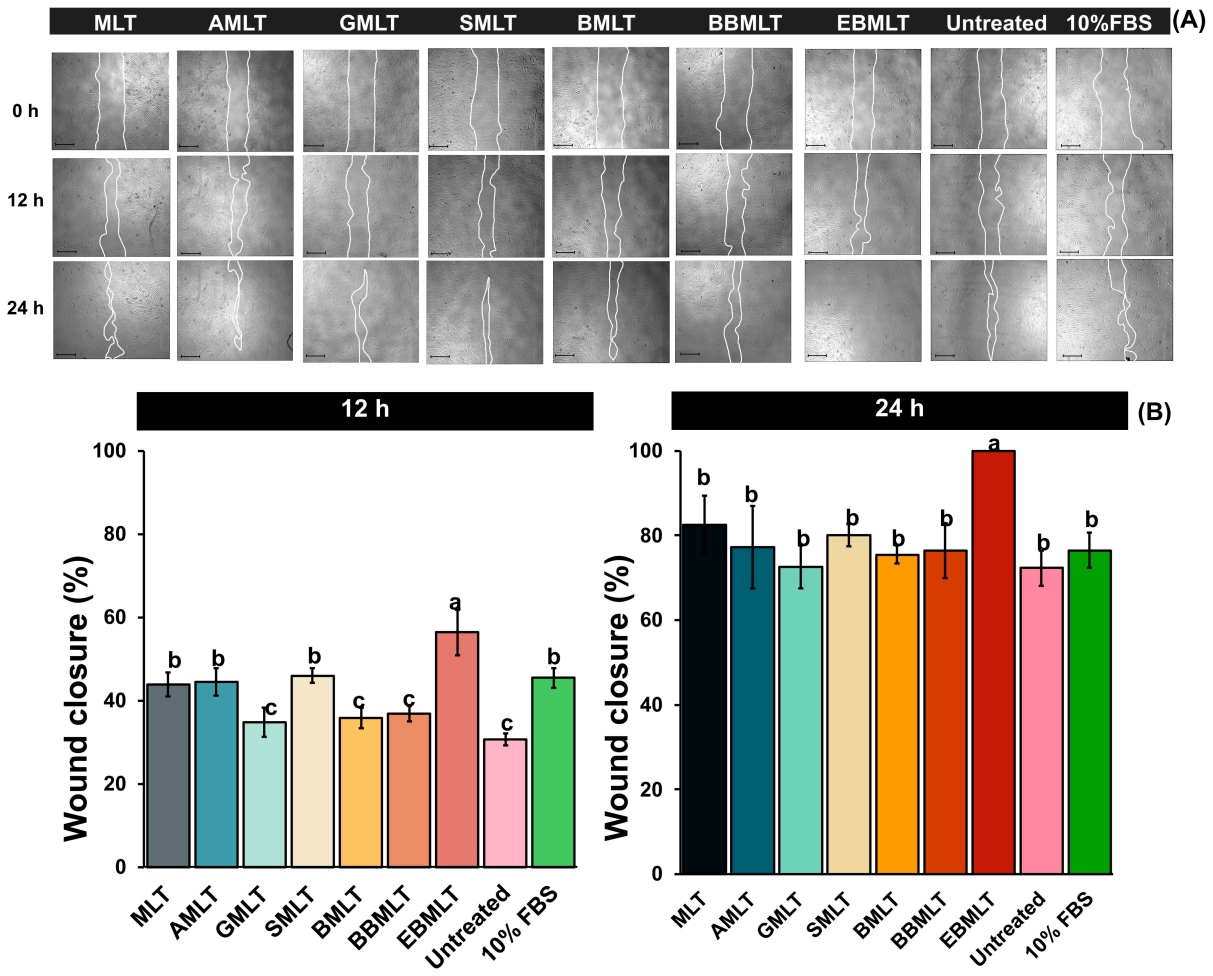
Images of wound closure (**A**) and wound closure scores in NHDF cells after incubating in 1 µM MLT and derivatives at 12 h and 24 h (**B**). Captured with 4X magnification with a scale bar of 650 µm at t = 0, 12, and 24 h, relative to wounds in healthy NHDF. Data indicated in mean ± SD (*n* = 5) by Tukey’s HSD; ^a–c^ means significant difference at *p* < 0.05.

To evaluate cell migration, a scratch assay was performed on the NHDF monolayer (Figure 18). After 12 h (Figure 18B), MLT, AMLT, SMLT, and EBMLT induced significantly faster wound closure compared to the untreated group (*p* < 0.001). Notably, EBMLT exhibited the most rapid closure rate at 56.45% within 12 h (*p* < 0.001), outperforming all other treatments, including the 10% FBS positive control, a standard growth supplement essential for typical cell development [51,52]. By 24 h, all treatment groups, including the untreated and 10% FBS controls, achieved over 70% wound closure (Figure 18B). Remarkably, EBMLT was the sole compound to achieve complete wound gap closure within 24 h. This outcome is consistent with our computational and SAR analyses, and aromatic-substituted MLT derivatives (BMLT, BBMLT, and EBMLT) tend to exhibit superior wound-healing activity compared to aliphatic-substituted compounds (AMLT, SMLT, and GMLT). Notably, the *N2*-bromobenzoyl MLT derivative, EBMLT, excels by enhancing both NHDF cell viability and migration.

Notably, a slight divergence was observed between the predicted computational binding affinities and the experimental assay results. While *N1*-aromatic substitution generally produced more favorable predicted binding affinity toward the selected targets, the *N2*-bromobenzoyl derivative (EBMLT) exhibited the most potent wound-closure activity in the NHDF scratch assay. This divergence highlights the importance of experimental validation beyond in silico predictions and suggests that computational binding and cellular activity should be interpreted as complementary rather than directly correlative.

Moreover, molecular studies and in vivo validation are required to provide additional mechanistic evidence, which we plan to address in future studies. These compelling benchmarks establish a solid foundation that warrants additional mechanistic evidence, which represents key objectives for subsequent investigations. To facilitate this clinical translation, we propose several crucial research avenues. First, studies should employ Western blotting, RT-qPCR, or enzyme-linked immunosorbent assays (ELISA) to quantitatively assess whether these derivatives actively inhibit or stimulate the expression and phosphorylation of these target proteins in NHDFs under both acute and chronic wound conditions. Second, since wound healing is a highly coordinated process involving multiple cell types, future work should expand the in vitro evaluation to multi-cellular models. This includes evaluating key derivatives on human keratinocytes (HaCaT) for re-epithelialization and human umbilical vein endothelial cells (HUVECs) to validate the predicted angiogenic effects via the VEGFR-1 pathway. Co-culture models would provide a more physiologically relevant representation of the wound microenvironment. Third, transitioning from in vitro models to in vivo animal studies is essential. In vivo evaluation will allow researchers to observe the systemic influence of these derivatives on granulation tissue formation, collagen deposition, and spatial angiogenesis in a complex, living tissue system. Lastly, to optimize the therapeutic efficacy of these hydrophobic MLT derivatives, the development of topical drug delivery systems should be explored. These formulations could provide sustained drug release at the wound site, paving the way for advanced dermatological applications in acute and chronic wound care.

## 3. Materials and Methods

### 3.1. Screening for Potential Target Genes of MLT and Its Derivatives

The Swiss Target Prediction (version 2023 release; http://swisstargetprediction.ch/, accessed on 22 March 2024) and SuperPred (version 3.0; https://prediction.charite.de/, accessed on 21 March 2025) databases are online tools for predicting drug targets. The SMILES formats of MLT and its derivatives were imported into the Swiss Target Prediction and SuperPred databases, and the species was set to “*Homo sapiens*”. The predictions of the potential targets of the active ingredients were obtained by using a probability >0.01 for Swiss target prediction and a probability >50% for SuperPred as the screening condition. Sequentially, the duplicated targets were deleted and the overlapping targets between Swiss target prediction and SuperPred were summarized by visualizing on a Venn diagram using VennPainter (version 1.2.0) [53].

### 3.2. Screening for Potential Wound Healing-Related Target Genes of MLT and Its Derivatives

A Venn diagram from VennPainter was utilized to summarize the potential targets of wound healing. The overlapping targets between Swiss Target Prediction and SuperPred databases were intersected with wound healing-related targets obtained from the GeneCards (version 5.24.0; https://www.genecards.org/, accessed on 5 May 2025) database. The input into the search box of the GeneCard database was “wound healing, hemostasis, inflammation, proliferation, remodeling”, and the species set as “*Homo sapiens*” to retrieve the related targets. After that, the targets associated with wound healing were identified as potential targets.

### 3.3. Network Pharmacology-Based Analysis of MLT and Derivatives on Wound Healing

PPI network analysis of the overlapping potential wound healing-related target genes was performed on the String database (https://cn.string-db.org/, accessed on 7 May 2025). The selected biological species was homo sapiens to obtain the PPI of MLT and its derivatives on wound-healing mechanisms. The provided PPI network data information was analyzed and visualized by Cytoscape (version 3.10.3). The PPI network data provided a 0.7 confidence score. Subsequently, the top ten potential target genes were analyzed by the CytoHubba (version 0.1) plug-in based on the degree method [54].

### 3.4. Pathway and Function Enrichment Analysis of MLT and Derivatives on Wound Healing

The ten probable wound healing-related targets list was analyzed on GeneMANIA (version 3.6.0; http://www.genemania.org, accessed on 16 March 2026), another server that interpolates several databases and technologies to anticipate and identify the relevant activities of individual genes in hub genes and establish gene priority and linkages. The interaction networks were predicted based on several criteria, including co-expression, physical interactions, shared protein domains, co-localization, shared pathways, and genetic interactions. Furthermore, enrichment pathways and related functions were analyzed via ShinyGO (version 0.85.1; https://bioinformatics.sdstate.edu/go/, accessed on 16 March 2026), and the statistical approach of input gene targets list on enrichment pathways and related functional annotations enrichment analysis were based on Kyoto Encyclopedia of Genes and Genomes (KEGG) [55] and gene ontology (GO) databases. The visualization of KEGG and GO was carried out by using SRplot (version, 2025 release; https://bioinformatics.com.cn/srplot, accessed on 27 November 2025) [56].

### 3.5. Identification of Differentially Expressed Genes (DEGs)

To find datasets reporting the data on expression levels of targets in wound healing, we searched the expression microarray datasets listed in the Gene Expression Omnibus (GEO) of the NCBI official website (online version, 2025 release; https://www.ncbi.nlm.nih.gov/geo/, accessed on 27 November 2025). The GSE268639 dataset was used. DEGs of non-healing wounds were visualized in a volcano plot by using SRplot (online version, 2025 release; https://bioinformatics.com.cn/srplot, accessed on 27 November 2025) [56].

### 3.6. Molecular Docking

The probable wound healing-related targets were studied on binding interaction with MLT and derivatives by using Autodock version 4.2 with AutodockTool version 1.5.7 [57]. The crystal structure of proteins is provided by the RCSB Protein Data Bank databases (https://www.rcsb.org/). The selected proteins, including NF-κB (PDB ID: 4KIK), EGFR (PDB ID: 7AEI), VEGFR (PDB ID: 3HNG), MMP-1 (PDB ID: 2TCL), and MMP-13 (PDB ID: 5B5O), were predicted to have the protonation state (at pH 7.4) by using PDB2QR (version 3.7.1, https://server.poissonboltzmann.org/pdb2pqr acceded on 11 December 2026) [58]. To validate the molecular docking simulations, the protein targets involved in wound healing responses, NF-κB, EGFR, VEGFR, ERK1, MMP-1, and MMP-13 were re-docked with their co-ligand inhibitors named KSA, R85, 8ST, SCH772984, RO4175, and WMM307, respectively. Their grid parameters were set to belong to each co-ligand’s attribution X × Y × Z by displaying in Table 2. After that, the validated molecular docking protocols were analyzed with MLT and derivatives. All ligands were prepared by Chem Draw Ultra 8.0 [59], then the protonation state of ligands was predicted at pH 7.4 [60], and the charge of protonated ligands was modified by Discovery Studio [61].

### 3.7. Biological Activity on Normal Human Dermal Cell

Normal human dermal fibroblast (NHDF) (ATCC, PCS-201-012, Manassas, VA, USA) was cultured with 10% fetal bovine serum (FBS) (Gibco, Grand Island, NY, USA) and 1.0% Penicillin/Streptomycin (Gibco, NY, USA) in Dulbecco’s Modified Eagle Medium (DMEM) (Gibco, NY, USA). NHDF cells were studied on biological activities associated with wound healing, including cell proliferation and etherealization effects. The 90% confluence NHDF cells were plated on to a 96-well plate with 5000 cells/well and incubated with completed media for 24 h. Then, cells were washed with phosphate-buffered saline (PBS) of pH 7.4 and replaced with MLT and derivatives at 0.1, 0.5, 1.0, 2.0, and 10.0 µM in FBS-free media. The FBS-free cultured media was the untreated group and the 10% FBS media was used as the positive (+) control group. The proliferation rates at 24 h were measured by using 3-(4,5-dimethylthiazol-2-yl)-2,5-diphenyltetrazolium bromide (MTT) assay; this protocol was following our previous publication [62]. Briefly, the MTT reagent at a concentration of 0.5 mg/mL and supernatant from tested samples were mixed in a ratio of 1:1 (*v*/*v*). The mixed test was incubated for 3 h, then released with DMSO, and measured at 570 nm. The results were analyzed with the following Equation (1).

Cell proliferation (%) = (A_tested_/A_untreated_) × 100 (1)

where A_tested_ refers to the absorbance of tested compounds or (+) control groups and A_untreated_ refers to the absorbance of the FBS-free media group.

Consequently, NHDF cells were studied for their epithelialization properties using a wound scratch assay, as described in our previous publication [62]. Cells were incubated into 24-well plates with 50,000 cells/well for 48 h. After that, cells were scratched by a 0.2 mL-tip then washed with PBS (pH 7.4) twice. The scratched cells were treated with 1.0 µM of MLT and all derivatives and captured initial wound area by using the EVOS™ FL Auto 2 Imaging System (Thermo Fisher Scientific, Waltham, MA, USA) at 4× magnification. The positive (+) control group was treated with 10% FBS media, and the untreated groups with FBS-free media. All samples were captured with the progression of wound closure at 12 and 24 h. The percentage of wound closure was analyzed through Equation (2).

Wound closure (%) = ((A_0_ − A_t_)/A_0_) × 100 (2)

where t refers to the time points at *n* = 0, 12, and 24 h.

### 3.8. Statistical Analysis

The biological activity in the cell-based assay was reported as mean ± SD (*n* = 5). The one-way analysis of variance with Tukey’s test using SPSS Statistics 28.0 (International Business Machines Corporation, Armonk, NY, USA) was used for data analysis. A post hoc analysis was used to classify groups based on mean values. A *p*-value < 0.05 was considered to indicate a statistically significant difference.

## 4. Conclusions

Our network pharmacology analysis indicated that MLT and its derivatives may influence wound healing through multiple mechanisms across the inflammatory, proliferative, and tissue-remodeling phases, with the HIF-1 signaling cascade identified as a bioinformatically prioritized candidate axis. The predicted SAR profile derived from molecular docking suggests that both the position and nature of the substituent influence receptor binding interactions, with aromatic substitution generally producing higher binding affinities toward targets associated with wound healing. Furthermore, the *N2*-bromobenzoyl-substituted derivative (EBMLT) displayed the greatest enhancement of NHDF cell proliferation and scratch-closure activity among the tested compounds. Overall, the present study provides a rational framework for the future design of MLT derivatives with improved biological activity and binding interactions for wound-healing applications.

## Figures and Tables

**Table 1:** The seven enriched functions from GeneMANIA analysis.

No	Functions	FDR	Genes	Gene Interactions
1	Cellular response to abiotic stimulus	4.27 × 10^−7^	9	**PIK3CA**, **NFKB1**, **MAPK3**, **MMP9**, **TLR4**, MYC, TNFRSF1A, STK11
2	Regulation of protein kinase B (AKT) signaling	1.29 × 10^−6^	8	**AKT1**, **EGFR**, **PIK3R1**, **PIK3CA**, PTEN, PIK3R2, IRS1, PHLPP1
3	Cellular response to oxidative stress	6.64 × 10^−6^	7	**AKT1**, **EGFR**, **MAPK3**, **MMP9**, TLR4, HIF1A, MYC, TNFFRSF1A, STK11
4	Regulation of epithelial cell proliferation	6.09 × 10^−5^	7	**AKT1**, **EGFR**, **TLR4**, **HIF1A**, ARNT, MYC, NF1
5	Cell cycle G1/S phase transition	6.62 × 10^−5^	7	**AKT1**, **EGFR**, AURKA, CCND2, CDKN3A, MYC, PTEN
6	Epithelial cell proliferation	1.11 × 10^−4^	7	**AKT1**, **EGFR**, **HIF1A**, **TLR4**, ARNT, MYC, NF1
7	Regulation of cyclin-dependent protein kinase activity	1.58 × 10^−4^	5	**AKT1**, **EGFR**, CDKN2A, CCND2, PTEN

Proteins corresponding to the genes indicated in bold text represent the top ten hub proteins.

**Table 2:** Validated protocol for ligand–protein interactions in wound healing study.

Protein	PDB	Co-Ligand	Grid Box (X, Y, Z)			Grid Side (X, Y, Z)			Grid Spacing	Binding Affinity
NF-κB	4KIK	KSA	48.38	30.06	−56.81	40	40	40	0.375	−11.92
EGFR	7AEI	R85	−0.63	50.62	−18.49	40	40	50	0.375	−9.72
VEGFR1	3HNG	8ST	4.70	17.80	33.40	40	40	50	0.375	−11.97
MMP-1	2TCL	RO4175	73.82	8.73	9.11	30	30	40	0.419	−8.00
MMP-13	5B5O	WMM307	10.53	13.68	19.30	35	30	30	0.375	−8.87

## Data Availability

The original contributions presented in this study are included in the article/Supplementary Materials. Further inquiries can be directed to the corresponding author.

## Supplementary Materials

The following supporting information can be downloaded at https://www.mdpi.com/article/10.3390/ijms27156699/s1.

## Abbreviations

The following abbreviations are used in this manuscript:

∆G	Gibbs free energy change
AMLT	*N*-(2-(1-acetyle-5-methoxy-1H-indol-3-yl)ethyl)acetamide
BMLT	*N*-(2-(1-benzoyle-5-metgoxy-1H-indol-3yl)ethyl)acetamide
BBMLT	*N*-(2-(1-4-bromobrnzoyl-5-metgoxy-1H-indol- 3yl)ethyl)acetamide
DEGs	differentially expressed genes
EBMLT	4-bromo-N-(2-(5-metgoxy-1H-indol-3yl)ethyl)benzamide
EGFR	epidermal growth factor receptor
ERK1	extracellular signal-regulated kinase 1
FDR	false discovery rate
GEO	gene expression omnibus
GMLT	*N*-(2-(1-glutaryl-5-methoxy-1H-indol-3-yl)ethyl)acetamide
GO	gene ontology
KEGG	Kyoto encyclopedia of genes and genomes
MMP-1	matrix metalloproteases 1
MMP-13	matrix metalloproteases 13
MLT	melatonin
RMSD	root means square deviation
SAR	Structure–activity relationship
SMLT	*N*-(2-(1-succinyl-5-methoxy-1H-indol-3-yl)ethyl)acetamide
VEGFR	vascular endothelial growth factor receptors
