# Supplementary material for: Structure–Activity Relationship Evaluation of Melatonin and Its Derivatives for Wound-Healing Applications: A Combined Network Pharmacology, Molecular Docking, and Biological Validation Approach
Source: Int J Mol Sci. 2026 Jul 27;27(15):6699. doi: 10.3390/ijms27156699 (PMC13467473; doi:10.3390/ijms27156699)
Supplement: Supplementary file 1 [file ijms-27-06699-s001.zip › ijms-4391108-supplementary.pdf]

Supplementary data

Table S1. Node degree of interaction enriches wound healing targets of MLT and its derivatives from PPI

Symbol	Full Protein Name	Ensembl Protein ID	UniProt	Degree
AKT1	RAC-alpha serine/threonine-protein kinase	9606.ENSP00000451828	P31749	48
EGFR	Epidermal growth factor receptor	9606.ENSP00000275493	P00533	45
MAPK3	Mitogen-activated protein kinase 3 (ERK1)	9606.ENSP00000263025	P27361	32
HIF1A	Hypoxia-inducible factor 1-alpha	9606.ENSP00000437955	Q16665	30
NFKB1	Nuclear factor NF-kappa-B p105 subunit	9606.ENSP00000226574	P19838	30
PIK3CA	Phosphatidylinositol 4,5-bisphosphate 3-kinase catalytic alpha	9606.ENSP00000263967	P42336	29
PIK3R1	Phosphatidylinositol 3-kinase regulatory subunit alpha	9606.ENSP00000428056	P27986	29
CASP3	Caspase-3	9606.ENSP00000311032	P42574	28
MMP9	Matrix metalloproteinase-9	9606.ENSP00000361405	P14780	28
TLR4	Toll-like receptor 4	9606.ENSP00000363089	O00206	28
MAPK1	Mitogen-activated protein kinase 1 (ERK2)	9606.ENSP00000215832	P28482	26
HSP90AB1	Heat shock protein HSP 90-beta	9606.ENSP00000360609	P08238	25
PIK3CB	Phosphatidylinositol 4,5-bisphosphate 3-kinase catalytic beta	9606.ENSP00000501150	P42338	25
ITGB1	Integrin beta-1	9606.ENSP00000379350	P05556	24
PIK3CD	Phosphatidylinositol 4,5-bisphosphate 3-kinase catalytic delta	9606.ENSP00000366563	O00329	24
STAT1	Signal transducer and activator of transcription 1-alpha/beta	9606.ENSP00000354394	P42224	24
PTGS2	Prostaglandin G/H synthase 2 (COX-2)	9606.ENSP00000356438	P35354	23
GRB2	Growth factor receptor-bound protein 2	9606.ENSP00000376345	P62993	22
FYN	Tyrosine-protein kinase Fyn	9606.ENSP00000346671	P06241	21
JAK2	Tyrosine-protein kinase JAK2	9606.ENSP00000371067	O60674	21
KDR	Vascular endothelial growth factor receptor 2	9606.ENSP00000263923	P35968	21
MTOR	Serine/threonine-protein kinase mTOR	9606.ENSP00000354558	P42345	21
PTPN11	Tyrosine-protein phosphatase non-receptor type 11	9606.ENSP00000489597	P35235	20
RHOA	Transforming protein RhoA	9606.ENSP00000400175	P61586	20
CDC42	Cell division control protein 42 homolog	9606.ENSP00000497733	P60953	19
ICAM1	Intercellular adhesion molecule 1	9606.ENSP00000264832	P05362	19
MAPK8	Mitogen-activated protein kinase 8 (JNK1)	9606.ENSP00000378974	P45983	19
RAF1	RAF proto-oncogene	9606.ENSP00000401888	P04049	19

Symbol	Full Protein Name	Ensembl Protein ID	UniProt	Degree
	serine/threonine-protein kinase			
PPARG	Peroxisome proliferator-activated receptor gamma	9606.ENSP00000287820	P37231	18
CASP8	Caspase-8	9606.ENSP00000351273	Q14790	17
PRKACA	cAMP-dependent protein kinase catalytic subunit alpha	9606.ENSP00000309591	P17612	17
PRKCA	Protein kinase C alpha type	9606.ENSP00000408695	P17252	17
ITGB2	Integrin beta-2	9606.ENSP00000380948	P05107	16
PRKCD	Protein kinase C delta type	9606.ENSP00000331602	P09174	16
VCAM1	Vascular cell adhesion protein 1	9606.ENSP00000294728	P19320	16
CXCR4	C-X-C chemokine receptor type 4	9606.ENSP00000386884	P61073	15
HDAC1	Histone deacetylase 1	9606.ENSP00000362649	Q13547	14
ITGB3	Integrin beta-3	9606.ENSP00000452786	P05106	14
PARP1	Poly [ADP-ribose] polymerase 1	9606.ENSP00000355759	P09874	14
CASP9	Caspase-9	9606.ENSP00000330237	P55211	13
PGR	Progesterone receptor	9606.ENSP00000325120	P06401	13
FLT1	Vascular endothelial growth factor receptor 1	9606.ENSP00000282397	P17948	12
PIK3CG	Phosphatidylinositol 4,5-bisphosphate 3-kinase catalytic gamma	9606.ENSP00000419260	P48736	12
MMP2	Matrix metalloproteinase-2	9606.ENSP00000219070	P08253	11
CDK2	Cyclin-dependent kinase 2	9606.ENSP00000266970	P24941	10
MAP3K7	Mitogen-activated protein kinase 7	9606.ENSP00000358335	O43318	10
NOS3	Nitric oxide synthase, endothelial	9606.ENSP00000297494	P29474	10
CASP1	Caspase-1	9606.ENSP00000433138	P29466	9
HDAC6	Histone deacetylase 6	9606.ENSP00000365804	Q9UBN7	9
ITGAL	Integrin alpha-L	9606.ENSP00000349252	P20333	9
ITGAV	Integrin alpha-V	9606.ENSP00000261023	P06756	9
MAP2K2	Dual specificity mitogen-activated protein kinase 2	9606.ENSP00000262948	P36507	9
MAPK13	Mitogen-activated protein kinase 13	9606.ENSP00000211287	O15264	9
PRKCQ	Protein kinase C theta type	9606.ENSP00000263125	Q04759	9
CHUK	Inhibitor of nuclear factor kappa-B kinase subunit alpha	9606.ENSP00000359424	O15111	8
ITK	Tyrosine-protein kinase ITK	9606.ENSP00000398655	Q08881	8
NFE2L2	Nuclear factor erythroid 2-related factor 2 (Nrf2)	9606.ENSP00000380252	Q16236	8
PSEN1	Presenilin-1	9606.ENSP00000326366	P49768	8
F2	Prothrombin	9606.ENSP00000308541	P00734	7
FGFR1	Fibroblast growth factor receptor 1	9606.ENSP00000393312	P11362	7
HDAC2	Histone deacetylase 2	9606.ENSP00000430432	Q92769	7
ITGA2B	Integrin alpha-IIb	9606.ENSP00000262407	P08514	7
LGALS3	Galectin-3	9606.ENSP00000254301	P17931	7
PRKCE	Protein kinase C epsilon type	9606.ENSP00000306124	Q02156	7
DPP4	Dipeptidyl peptidase 4	9606.ENSP00000353731	P27487	6
KEAP1	Kelch-like ECH-associated protein 1	9606.ENSP00000377245	Q14164	6

Symbol	Full Protein Name	Ensembl Protein ID	UniProt	Degree
MPO	Myeloperoxidase	9606.ENSP00000225275	P05164	6
NOS2	Nitric oxide synthase, inducible	9606.ENSP00000327251	P35228	6
NTRK1	High affinity nerve growth factor receptor	9606.ENSP00000431418	P04629	6
NTRK2	BDNF/NT-3 growth factors receptor	9606.ENSP00000277120	P15289	6
PPARA	Peroxisome proliferator-activated receptor alpha	9606.ENSP00000385523	Q07869	6
SLC9A1	Sodium/hydrogen exchanger 1	9606.ENSP00000263980	P19634	6
AGTR1	Type-1 angiotensin II receptor	9606.ENSP00000398832	P30556	5
AXL	Tyrosine-protein kinase receptor UFO	9606.ENSP00000301178	P30530	5
CDC25C	M-phase inducer phosphatase 3	9606.ENSP00000321656	P30307	5
CDK5	Cyclin-dependent kinase 5	9606.ENSP00000419782	P49615	5
MME	Neprilysin	9606.ENSP00000418525	P08473	5
MMP1	Interstitial collagenase (MMP-1)	9606.ENSP00000322788	P03956	5
MMP3	Stromelysin-1 (MMP-3)	9606.ENSP00000299855	P08254	5
NOS1	Nitric oxide synthase, brain	9606.ENSP00000477999	P29475	5
PLA2G2A	Phospholipase A2, membrane associated	9606.ENSP00000383364	P14555	5
PRKCI	Protein kinase C iota type	9606.ENSP00000295797	P41743	5
PTGS1	Prostaglandin G/H synthase 1 (COX-1)	9606.ENSP00000354612	P23219	5
REN	Renin	9606.ENSP00000272190	P00797	5
SELE	E-selectin	9606.ENSP00000331736	P16581	5
ALOX12	Arachidonate 12-lipoxygenase	9606.ENSP00000251535	P18054	4
ALOX5	Arachidonate 5-lipoxygenase	9606.ENSP00000363512	P09917	4
CTSG	Cathepsin G	9606.ENSP00000216336	P08311	4
NOX1	NADPH oxidase 1	9606.ENSP00000362057	Q9Y5S8	4
NR3C2	Mineralocorticoid receptor	9606.ENSP00000350815	P08235	4
P2RX7	P2X purinoceptor 7	9606.ENSP00000330696	Q99572	4
P2RY12	P2Y purinoceptor 12	9606.ENSP00000307259	Q9H244	4
PIK3C2A	Phosphatidylinositol 4-phosphate 3-kinase catalytic alpha	9606.ENSP00000265970	O00443	4
PLAT	Tissue-type plasminogen activator	9606.ENSP00000220809	P00750	4
PLD1	Phospholipase D1	9606.ENSP00000342793	Q13393	4
SLC6A4	Sodium-dependent serotonin transporter	9606.ENSP00000261707	P31645	4
GRK2	Beta-adrenergic receptor kinase 1	9606.ENSP00000312262	P25098	3
GZMB	Granzyme B	9606.ENSP00000216341	P10144	3
HDAC9	Histone deacetylase 9	9606.ENSP00000408617	Q9UKV0	3
HMGCR	HMG-CoA reductase	9606.ENSP00000287936	P04035	3
KLF5	Krueppel-like factor 5	9606.ENSP00000366915	Q13887	3
PKM	Pyruvate kinase PKM	9606.ENSP00000320171	P14618	3
SCD	Stearoyl-CoA desaturase	9606.ENSP00000359380	O00767	3
ACHE	Acetylcholinesterase	9606.ENSP00000303211	P22303	2
APEX1	DNA-(apurinic or apyrimidinic site) lyase	9606.ENSP00000216714	P27695	2
CHKA	Choline kinase alpha	9606.ENSP00000265689	P35790	2

Symbol	Full Protein Name	Ensembl Protein ID	UniProt	Degree
HNF4A	Hepatocyte nuclear factor 4-alpha	9606.ENSP00000312987	P41235	2
KDM1A	Lysine-specific histone demethylase 1A	9606.ENSP00000383042	O60341	2
NOX4	NADPH oxidase 4	9606.ENSP00000263317	Q9NPH5	2
NR1I2	Nuclear receptor subfamily 1 group I member 2 (PXR)	9606.ENSP00000336528	O75469	2
P2RY1	P2Y purinoceptor 1	9606.ENSP00000304767	P47900	2
PDE4A	cAMP-specific 3',5'-cyclic phosphodiesterase 4A	9606.ENSP00000370078	P27815	2
PRCP	Lysosomal Pro-X carboxypeptidase	9606.ENSP00000377055	P42785	2
PTGIS	Prostacyclin synthase	9606.ENSP00000244043	Q16647	2
S100B	Protein S100-B	9606.ENSP00000291700	P04271	2
SHH	Sonic hedgehog protein	9606.ENSP00000297261	Q15465	2
TFPI	Tissue factor pathway inhibitor	9606.ENSP00000233156	P10646	2
ULK1	Serine/threonine-protein kinase ULK1	9606.ENSP00000324560	O75385	2
ADAM10	Disintegrin and metalloproteinase protein 10	9606.ENSP00000260408	O14672	1
ADRA2A	Alpha-2A adrenergic receptor	9606.ENSP00000280155	P08913	1
AOC3	Membrane primary amine oxidase	9606.ENSP00000312326	Q16853	1
BCHE	Cholinesterase (Butyrylcholinesterase)	9606.ENSP00000264381	P06276	1
BDKRB1	B1 bradykinin receptor	9606.ENSP00000216629	P46663	1
CETP	Cholesteryl ester transfer protein	9606.ENSP00000200676	P11597	1
CTSD	Cathepsin D	9606.ENSP00000236671	P07339	1
CYP3A4	Cytochrome P450 3A4	9606.ENSP00000498939	P08684	1
F13A1	Coagulation factor XIII A chain	9606.ENSP00000264870	P00488	1
HTR2A	5-hydroxytryptamine receptor 2A	9606.ENSP00000437737	P28223	1
KIF11	Kinesin-like protein KIF11	9606.ENSP00000260731	P52732	1
MERTK	Tyrosine-protein kinase Mer	9606.ENSP00000295408	Q12866	1
PDE2A	cGMP-dependent 3',5'-cyclic phosphodiesterase	9606.ENSP00000334910	O00408	1
PDE4D	cAMP-specific 3',5'-cyclic phosphodiesterase 4D	9606.ENSP00000345502	Q08499	1
C5AR1	C5a anaphylatoxin chemotactic receptor 1	9606.ENSP00000347197	P21730	0
DDR2	Discoidin domain-containing receptor 2	9606.ENSP00000356899	Q16832	0
HPSE	Heparanase	9606.ENSP00000384262	Q9Y251	0
HTR4	5-hydroxytryptamine receptor 4	9606.ENSP00000353915	Q13639	0
NAMPT	Nicotinamide phosphoribosyl transferase	9606.ENSP00000222553	P43490	0
NR1H2	Oxysterols receptor LXR-beta	9606.ENSP00000499121	P55055	0
PAM	Peptidyl-glycine alpha-amidating monooxygenase	9606.ENSP00000306100	P19021	0
PIN1	Peptidyl-prolyl cis-trans isomerase NIMA-interacting 1	9606.ENSP00000247970	Q13526	0
S1PR3	Sphingosine 1-phosphate receptor 3	9606.ENSP00000365006	Q9H228	0

Symbol	Full Protein Name	Ensembl Protein ID	UniProt	Degree
TACR1	Substance-P receptor	9606.ENSP00000303522	P25103	0
TAOK1	Serine/threonine-protein kinase TAO1	9606.ENSP00000261716	Q9P2K8	0
TBXA2R	Thromboxane A2 receptor	9606.ENSP00000393333	P21708	0
TMPRSS6	Transmembrane protease serine 6	9606.ENSP00000384964	Q8IU80	0

Table S2. Biological process of ten potential proteins on wound healing

No	Biological process	Enrichment FDR	Fold Enrichment	Genes	Genes list
1	Cellular response to abiotic stimulus	8.91×10^{-11}	52.41239	8	MMP9, PIK3CA, PIK3R1, EGFR, CASP3, MAPK3, NFKB1, TLR4
2	Cellular response to environmental stimulus	8.91×10^{-11}	52.41239	8	MMP9, PIK3CA, PIK3R1, EGFR, CASP3, MAPK3, NFKB1, TLR4
3	Response to abiotic stimulus	8.91×10^{-11}	19.34942	10	HIF1A, MMP9, AKT1, EGFR, NFKB1, PIK3CA, PIK3R1, CASP3, MAPK3, TLR4
4	Pos. reg. of smooth muscle cell proliferation	6.09×10^{-10}	138.1663	6	AKT1, PIK3CA, TLR4, EGFR, MMP9, PIK3R1
5	Response to oxygen-containing compound	2.62×10^{-9}	13.12528	10	TLR4, PIK3R1, HIF1A, MMP9, PIK3CA, AKT1, EGFR, MAPK3, NFKB1, CASP3
6	Reg. of smooth muscle cell proliferation	3.86×10^{-9}	89.45385	6	AKT1, PIK3CA, TLR4, EGFR, MMP9, PIK3R1
7	Cellular response to chemical stress	3.86×10^{-9}	47.32733	7	HIF1A, MMP9, PIK3CA, CASP3, MAPK3, AKT1, EGFR
8	Cell surface receptor protein tyrosine kinase signaling pathway	3.86×10^{-9}	28.49372	8	PIK3R1, HIF1A, AKT1, EGFR, CASP3, MAPK3, PIK3CA, MMP9
9	Response to nitrogen compound	3.86×10^{-9}	18.12312	9	PIK3R1, PIK3CA, TLR4, AKT1, MMP9, MAPK3, NFKB1, EGFR, CASP3
10	Smooth muscle cell proliferation	4.04×10^{-9}	87.2175	6	AKT1, PIK3CA, TLR4, EGFR, MMP9, PIK3R1
11	Cellular response to oxygen-containing compound	7.08×10^{-9}	16.40455	9	PIK3R1, MMP9, PIK3CA, TLR4, AKT1, EGFR, HIF1A, MAPK3, NFKB1
12	Apoptotic proc.	7.08×10^{-9}	10.97075	10	EGFR, CASP3, HIF1A, AKT1, MMP9 MAPK3, NFKB1, PIK3CA, TLR4, PIK3R1
13	Cellular response to endogenous stimulus	7.20×10^{-9}	16.18886	9	PIK3R1, HIF1A, PIK3CA, AKT1, CASP3, MAPK3, TLR4, EGFR, NFKB1
14	Reg. of localization	7.20×10^{-9}	10.69825	10	NFKB1, MMP9, PIK3CA, AKT1, PIK3R1, EGFR, HIF1A, MAPK3, CASP3, TLR4
15	Programmed cell death	7.20×10^{-9}	10.58625	10	EGFR, CASP3, HIF1A, AKT1, MMP9, MAPK3, NFKB1, PIK3CA, TLR4, PIK3R1
16	Cell death	7.20×10^{-9}	10.56701	10	EGFR, CASP3, HIF1A, AKT1, MMP9, MAPK3, NFKB1, PIK3CA, TLR4, PIK3R1
17	Response to reactive oxygen species	1.13×10^{-8}	67.41449	6	HIF1A, MMP9, CASP3, MAPK3, AKT1, EGFR
18	Cellular response to chemical stimulus	1.51×10^{-8}	9.698916	10	HIF1A, PIK3R1, MMP9, PIK3CA, TLR4, AKT1, EGFR, MAPK3, NFKB1, CASP3
19	Muscle cell proliferation	1.67×10^{-8}	62.02133	6	AKT1, PIK3CA, TLR4, EGFR, MMP9,

No	Biological process	Enrichment FDR	Fold Enrichment	Genes	Genes list
					PIK3R1
20	Response to endogenous stimulus	2.67×10^{-8}	13.36667	9	PIK3R1, HIF1A, MAPK3, PIK3CA, TLR4, AKT1, CASP3, EGFR, NFKB1

Table S3. Cellular component of ten potential proteins on wound healing

No	Cellular component	Enrichment FDR	Fold Enrichment	Genes	Genes list
1	Phosphatidylinositol 3-kinase complex class ia	4.22 × 10-4	516.8444	2	PIK3CA, PIK3R1
2	Phosphatidylinositol 3-kinase complex class i	4.22 × 10-4	516.8444	2	PIK3CA, PIK3R1
3	Cell leading edge	1.06 × 10-3	20.44659	4	EGFR, PIK3CA, TLR4, AKT1
4	Anchoring junction	1.06 × 10-3	11.6757	5	EGFR, MAPK3, PIK3CA, AKT1, PIK3R1
5	Phosphatidylinositol 3-kinase complex	2.01 × 10-3	155.0533	2	PIK3CA, PIK3R1
6	Glutamatergic synapse	2.01 × 10-3	15.30132	4	MAPK3, AKT1, EGFR, CASP3
7	Perinuclear region of cytoplasm	3.54 × 10-3	11.70214	4	TLR4, PIK3R1, EGFR, PIK3CA
8	Membrane protein complex	3.54 × 10-3	7.789015	5	PIK3CA, PIK3R1, CASP3, TLR4, EGFR
9	Plasma membrane bounded cell projection	3.54 × 10-3	5.698163	6	EGFR, HIF1A, MAPK3, PIK3CA, TLR4 AKT1
10	Cell projection	3.54 × 10-3	5.425661	6	EGFR, HIF1A, MAPK3, PIK3CA, TLR4, AKT1
11	Cell junction	3.5 × 10-3	5.356929	6	EGFR, MAPK3, PIK3CA, AKT1, PIK3R1, CASP3
12	Early endosome	1.48 × 10-3	13.14011	3	EGFR, MAPK3, TLR4
13	Phosphatidylinositol 3-kinase complex class ib	1.51 × 10-3	581.45	1	PIK3CA
14	I-kappab/nf-kappab complex	1.51 × 10-3	581.45	1	NFKB1
15	Perinuclear endoplasmic reticulum membrane	1.51 × 10-3	581.45	1	PIK3R1
16	Cell-cell junction	1.51 × 10-3	12.66316	3	PIK3CA, AKT1, PIK3R1
17	Lipopolysaccharide receptor complex	1.68 × 10-3	465.16	1	TLR4
18	Nf-kappab complex	1.68 × 10-3	465.16	1	NFKB1
19	Multivesicular body internal vesicle	1.91 × 10-2	387.6333	1	EGFR
20	Ruffle	2.02 × 10-2	23.85436	2	EGFR, TLR4

Table S4. Molecular function of ten potential proteins on wound healing

No	Molecular function	Enrichment FDR	Fold Enrichment	Genes	Genes list
1	Kinase regulator activity	1.76×10^{-5}	37.87948	5	PIK3R1, EGFR, PIK3CA, CASP3, AKT1
2	Protein kinase regulator activity	3.16×10^{-4}	34.97444	4	EGFR, PIK3CA, CASP3, AKT1
3	Molecular function activator activity	3.62×10^{-4}	10.56382	6	CASP3, AKT1, EGFR, PIK3CA, PIK3R1, HIF1A
4	Phosphotransferase activity alcohol group as acceptor	3.62×10^{-4}	15.48469	5	AKT1, MAPK3, PIK3CA, EGFR, PIK3R1
5	Insulin receptor substrate binding	4.14×10^{-4}	357.8154	2	PIK3CA, PIK3R1
6	Kinase activity	4.14×10^{-4}	13.86055	5	AKT1, MAPK3, PIK3CA, EGFR, PIK3R1
7	Kinase activator activity	7.24×10^{-4}	46.516	3	EGFR, PIK3CA, PIK3R1
8	Protein serine/threonine kinase activity	7.24×10^{-4}	19.96395	4	AKT1, MAPK3, PIK3CA, EGFR
9	Transferase activity transferring phosphorus-containing groups	7.24×10^{-4}	11.35645	5	AKT1, MAPK3, PIK3CA, EGFR, PIK3R1
10	Phosphatidylinositol kinase activity	1.10×10^{-3}	172.2815	2	PIK3CA, PIK3R1
11	Phosphatase binding	1.62×10^{-3}	31.42973	3	EGFR, MAPK3, PIK3R1
12	Protein kinase activity	1.62×10^{-3}	14.62767	4	AKT1, MAPK3, EGFR, PIK3CA
13	Enzyme activator activity	1.70×10^{-3}	14.1386	4	CASP3, EGFR, PIK3CA, PIK3R1
14	serine/threonine kinase inhibitor activity	1.77×10^{-3}	110.7524	2	CASP3, AKT1
15	Lipid kinase activity	1.77×10^{-3}	108.1767	2	PIK3CA, PIK3R1
16	Enzyme binding	1.77×10^{-3}	6.077875	6	HIF1A, AKT1, EGFR, CASP3, MAPK3, PIK3R1
17	Phosphotyrosine residue binding	1.85×10^{-3}	96.90833	2	MAPK3, PIK3R1
18	Protein kinase binding	1.85×10^{-3}	12.20892	4	AKT1, EGFR, HIF1A, PIK3R1
19	Enzyme regulator activity	1.85×10^{-3}	7.884068	5	PIK3R1, CASP3, EGFR, PIK3CA AKT1
20	Identical protein binding	1.85×10^{-3}	5.747446	6	AKT1, NFKB1, MMP9, MAPK3 TLR4, EGFR

Table S5. KEGG pathway enrichment of ten potential proteins on wound healing

No	Pathway	Enrichment FDR	Fold Enrichment	Genes	Genes list
1	PD-L1 expression and PD-1 checkpoint pathway in cancer	2.05×10^{-16}	206.74	8	EGFR, AKT1, HIF1A, NFKB1, PIK3CA, PIK3R1, MAPK3, TLR4
2	Proteoglycans in cancer	2.05×10^{-16}	103.11	9	EGFR, AKT1, HIF1A, MMP9, PIK3CA, PIK3R1, MAPK3, TLR4, CASP3
3	HIF-1 signaling pathway	4.48×10^{-16}	170.70	8	EGFR, AKT1, HIF1A, NFKB1, PIK3CA, PIK3R1, MAPK3, TLR4
4	Hepatitis B	9.14×10^{-15}	114.15	8	AKT1, MMP9, NFKB1, PIK3CA, PIK3R1, MAPK3, TLR4, CASP3
5	Prostate cancer	5.85×10^{-14}	167.84	7	EGFR, AKT1, MMP9, NFKB1, PIK3CA, PIK3R1, MAPK3
6	Lipid and atherosclerosis	5.85×10^{-14}	86.54	8	AKT1, MMP9, NFKB1, PIK3CA, PIK3R1, MAPK3, TLR4, CASP3
7	TNF signaling pathway	2.18×10^{-13}	136.81	7	AKT1, MMP9, NFKB1, PIK3CA, PIK3R1, MAPK3, CASP3
8	Relaxin signaling pathway	3.03×10^{-13}	126.21	7	EGFR, AKT1, MMP9, NFKB1, PIK3CA, PIK3R1, MAPK3
9	Pathways in cancer	3.03×10^{-13}	39.57	9	EGFR, AKT1, HIF1A, MMP9, NFKB1, PIK3CA, PIK3R1, MAPK3, CASP3
10	Hepatitis C	1.21×10^{-12}	102.39	7	EGFR, AKT1, NFKB1, PIK3CA, PIK3R1, MAPK3, CASP3
11	Influenza A	1.85×10^{-12}	95.21	7	AKT1, NFKB1, PIK3CA, PIK3R1, MAPK3, TLR4, CASP3
12	Central carbon metabolism in cancer	1.90×10^{-12}	196.55	6	EGFR, AKT1, HIF1A, PIK3CA, PIK3R1, MAPK3
13	Pancreatic cancer	2.90×10^{-12}	181.23	6	EGFR, AKT1, NFKB1, PIK3CA, PIK3R1, MAPK3
14	Kaposi sarcoma-associated herpesvirus infection	3.55×10^{-12}	83.92	7	AKT1, HIF1A, NFKB1, PIK3CA, PIK3R1, MAPK3, CASP3
15	Colorectal cancer	5.34×10^{-12}	160.40	6	EGFR, AKT1, PIK3CA, PIK3R1, MAPK3, CASP3
16	Human immunodeficiency virus 1 infection	5.45×10^{-12}	77.53	7	AKT1, NFKB1, PIK3CA, PIK3R1, MAPK3, TLR4, CASP3
17	Endocrine resistance	7.96×10^{-12}	145.36	6	EGFR, AKT1, MMP9, PIK3CA, PIK3R1, MAPK3
18	Human cytomegalovirus infection	7.96×10^{-12}	72.68	7	EGFR, AKT1, NFKB1, PIK3CA, PIK3R1, MAPK3, CASP3

No	Pathway	Enrichment FDR	Fold Enrichment	Genes	Genes list
19	Chemical carcinogenesis-reactive oxygen species	7.96×10^{-12}	71.72	7	EGFR, AKT1, HIF1A, NFKB1, PIK3CA, PIK3R1, MAPK3
20	AGE-RAGE signaling pathway in diabetic complications	8.45×10^{-12}	140.96	6	AKT1, NFKB1, PIK3CA, PIK3R1, MAPK3, CASP3

Table S6. Degree of interaction enrich of wound healing targets of MLT and its derivatives from PPI

Symbol	Full Protein Name	Node Degree
AKT1	RAC-alpha serine/threonine-protein kinase	9
EGFR	Epidermal growth factor receptor	8
TLR4	Toll-like receptor 4	8
CASP3	Caspase-3	7
MMP9	Matrix metalloproteinase-9	7
NFKB1	Nuclear factor NF-kappa-B p105 subunit	7
HIF1A	Hypoxia-inducible factor 1-alpha	6
MAPK3	Mitogen-activated protein kinase 3 (ERK1)	6
PIK3CA	Phosphatidylinositol 4,5-bisphosphate 3-kinase catalytic alpha	4
PIK3R1	Phosphatidylinositol 3-kinase regulatory subunit alpha	4

Table S7. The target related of MLT and derivatives

Compound	Target number	Target list
MLT	6	EGFR, HIF1A, MMP9, NFKB1, PI3KR1, PIK3CA
AMLT	4	EGFR, HIF1A, NFKB1, PI3KR1
BMLT	4	EGFR, HIF1A, PI3KR1, TLR4
BBMLT	3	CASP3, EGFR, TLR4
EBMLT	4	AKT1, EGFR, MAPK3, PI3KR1
SMLT	5	CASP3, EGFR, HIF1A, PI3KR1, TLR4
GMLT	6	EGFR, CASP3, HIF1A, NFKB1, PIK3CA, TLR4

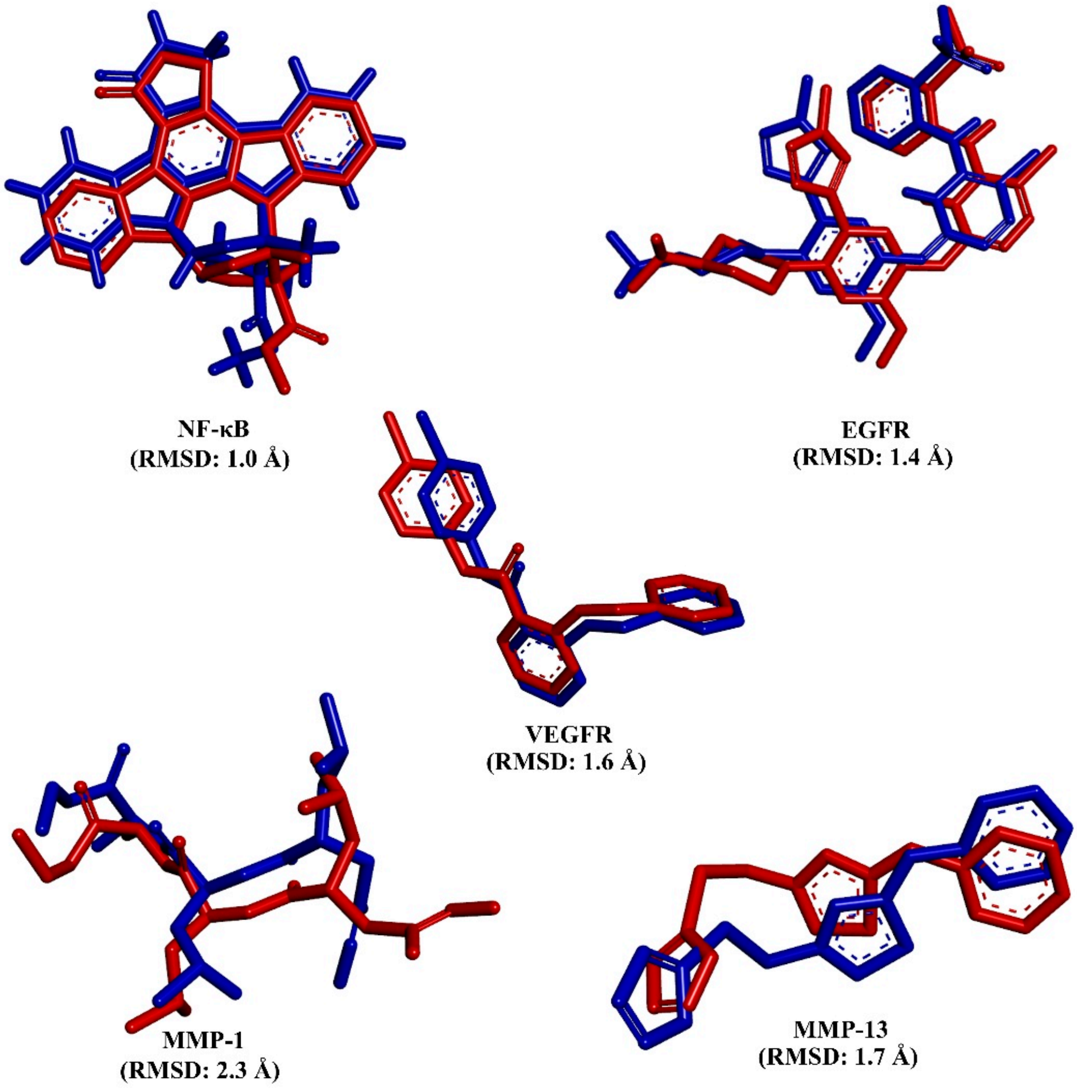

Figure S1. The supervision of co-ligand between original and re-docking of all selected proteins. Red means co-ligand from database and blue means co-ligand from re-docking.

Table S8. The binding interactions of MLT and its derivatives on NF-κB protein.

Compound	H-bond (Å)	Hydrophobic interactions
MLT	C99 (1.71), D166 (2.03)	V29, A42, K44, L94, M96, I165
AMLT	C99 (1.71), D166 (1.99)	V29, A42, K44, M65, L94, M96, I165
BMLT	C99 (2.12), K106 (2.17)	L21, A42, V74, K106, V152, I165
BBMLT	C99 (1.99), L167 (2.60)	L21, V29, A42, G102, K106, V152, I165
EBMLT	M96 (2.99), D166 (1.65)	L21, V29, A42, K44, C99, V152, I165
SMLT	-	L21, A42, V74, C99, V152, I165
GMLT	C99 (2.06), K106 (1.75)	L21, A42, V74, C99, V152, I165

Table S9. The binding interactions of MLT and its derivatives on EGFR from molecular docking simulation

Compound	H-bond (Å)	Hydrophobic interactions
MLT	M793 (1.85)	L718, L844, A743, M793
AMLT	K745 (1.73, 1.85), M793 (1.70)	V726, L718, A743, M766, C775, L844
SMLT	K745 (1.86), M793 (2.05), C797 (1.87)	L718, V726, A743, L792, L844
GMLT	K745 (2.41), M793 (1.99), C797 (1.86)	L718, L792, L844
BMLT	K745 (1.77, 2.09), M793 (1.78)	L718, F723, V726, A743, M766, C775, L844
BBMLT	K716 (1.82), M793 (1.82)	L718, A743, M766, C775, C797, L844
EBMLT	K745 (1.69), M793 (1.80)	V726, A743, M766, C775, L844

Table S10. The binding interactions of MLT and its derivatives on VGFR from molecular docking simulation

Compound	H-bond (Å)	Hydrophobic interactions
MLT	K432 (2.04), N447 (1.99)	L401, V434, A439
AMLT	C912 (1.86)	A859, L882, V891, V892, V909
SMLT	C912 (2.71)	V841, A859, K861, L882, V907, V909
GMLT	C912 (2.83)	V841, A859, K861, V892, V907, V909
BMLT	K861 (2.38), C912 (2.17)	V841, A859, K861, V892, V907, V909, C1039
BBMLT	V892 (2.66)	K861, L882, V892, V909
EBMLT	-	L833, V841, A859, I881, L882, V892, C912, V909, Y991, L1029, C1039, F1041

Table S11. Binding interaction of MLT and six derivatives with MMP-1 from molecular docking simulation

Compound	H-bond (Å)	Hydrophobic interactions
MLT	Y140 (1.69), T141 (2.64)	L81, A82
AMLT	R114 (2.71), T141 (2.34)	L81, H118
SMLT	R114 (2.31), S139 (1.81), Y140 (1.79)	L81, H118, H122
GMLT	A84 (2.10), R114 (1.80), L135 (3.07), Y137 (3.03), Y140 (3.01), T141 (2.24)	L81
BMLT	R114 (2.63)	L81, Y110, H118, H128
BBMLT	A84 (1.78), E119 (2.88)	L81, Y110, R114, V115, H118, H122, Y140
EBMLT	-	L81, Y110, R114, V115, H118, Y140

Table S12. Binding interaction of MLT and six derivatives with MMP-13 from molecular docking simulation

Compound	H-bond (Å)	Hydrophobic interactions
MLT	Y244 (1.94), Y245 (2.14)	V219, H222, Y244
AMLT	T245 (2.07)	L185, Y214, H222
SMLT	A188 (1.90), E223 (2.24), T245 (2.14)	L185, Y214
GMLT	A188 (2.00), E223 (2.27), T245 (2.06)	L185, Y214
BMLT	L185 (2.02), A186 (2.11)	L184, L185, A186, V219, H222
BBMLT	A188 (2.01), E223 (1.74)	L185, Y214, V219, H222, H226, Y244
EBMLT	L185 (1.82), A186 (2.43)	V219, H222, H226, Y244
